# Synthesis and Evaluation of Novel *S*-alkyl Phthalimide- and *S*-benzyl-oxadiazole-quinoline Hybrids as Inhibitors of Monoamine Oxidase and Acetylcholinesterase

**DOI:** 10.3390/ph16010011

**Published:** 2022-12-22

**Authors:** Bilal Ahmad Khan, Syeda Shamila Hamdani, Saquib Jalil, Syeda Abida Ejaz, Jamshed Iqbal, Ahmed M. Shawky, Alaa M. Alqahtani, Gamal A. Gabr, Mahmoud A. A. Ibrahim, Peter A. Sidhom

**Affiliations:** 1 Department of Chemistry, University of Azad Jammu and Kashmir, Muzaffarabad 13100, Pakistan; 2 Center for Advanced Drug Research, COMSATS University Islamabad, Abbottabad Campus, Abbottabad 22060, Pakistan; 3 Department of Pharmaceutical Chemistry, Faculty of Pharmacy, The Islamia University of Bahawalpur, Bahawalpur 63100, Pakistan; 4 Science and Technology Unit (STU), Umm Al-Qura University, Makkah 21955, Saudi Arabia; 5 Pharmaceutical Chemistry Department, College of Pharmacy, Umm Al-Qura University, Makkah 21955, Saudi Arabia; 6 Department of Pharmacology and Toxicology, College of Pharmacy, Prince Sattam Bin Abdulaziz University, Al-Kharj 11942, Saudi Arabia; 7 Agricultural Genetic Engineering Research Institute (AGERI), Agricultural Research Center, Giza 12619, Egypt; 8 Computational Chemistry Laboratory, Chemistry Department, Faculty of Science, Minia University, Minia 61519, Egypt; 9 School of Health Sciences, University of KwaZulu-Natal, Westville, Durban 4000, South Africa; 10 Department of Pharmaceutical Chemistry, Faculty of Pharmacy, Tanta University, Tanta 31527, Egypt

**Keywords:** oxadiazole-quinoline hybrids, Alzheimer’s illness, monoamine oxidase, AChE, molecular modeling

## Abstract

New *S*-alkyl phthalimide **5a**–**f** and *S*-benzyl **6a**–**d** analogs of 5-(2-phenylquinolin-4-yl)-1,3,4-oxadiazole-2-thiol (**4**) were prepared by reacting **4** with *N*-bromoalkylphthalimide and CF_3_-substituted benzyl bromides in excellent yields. Spectroscopic techniques were employed to elucidate the structures of the synthesized molecules. The inhibition activity of newly synthesized molecules toward MAO-A, MAO-B, and AChE enzymes, was also assessed. All these compounds showed activity in the submicromolar range against all enzymes. Compounds **5a** and **5f** were found to be the most potent compounds against MAO-A (IC_50_ = 0.91 ± 0.15 nM) and MAO-B (IC_50_ = 0.84 ± 0.06 nM), while compound **5c** showed the most efficient acetylcholinesterase inhibition (IC_50_ = 1.02± 0.65 μM). Docking predictions disclosed the docking poses of the synthesized molecules with all enzymes and demonstrated the outstanding potency of compounds **5a**, **5f**, and **5c** (docking scores = −11.6, −15.3, and −14.0 kcal/mol against MAO-A, MAO-B, and AChE, respectively). These newly synthesized analogs act as up-and-coming candidates for the creation of safer curative use against Alzheimer’s illness.

## 1. Introduction

Alzheimer’s disease (AD) is a complex neurological disturbance associated with memory loss and language skills, as well as behavioral and psychological changes [1]. According to the Alzheimer’s Association report, about 0.47 billion people globally suffer from AD, and the number of patients will exceed 1.50 billion in 2050 [2]. AD is a multi-factorial pathological condition with several causes. Among these, the deposition of beta-amyloid fibers, the death of neural cells, and high levels of monoamine oxidase (MAO) and acetylcholinesterase (AChE) enzymes occur [3,4].

The MAO enzyme presents in the exterior membrane of mitochondria [5] and metabolizes a variety of dietary amines besides neurotransmitters [6]. There are two known variants of the MAO enzyme, MAO-A and MAO-B, which have 70% similarity in protein structure and can be found in different parts of the body, liver, and brain [7]. MAO-B catalyzes beta-phenylethylamine and benzylamine, while MAO-A preferably catalyzes serotonin, noradrenaline, and adrenaline [5]. The inhibition of these enzymes plays a key role in AD [8,9]. In addition to MAOs, the AChE enzyme is a target for AD management [10,11]. The inhibition of these neural enzymes (AChE and MAOs) possess neuroprotective effects and decreases oxidative stress [12], leading to an increased level of neurotransmitters in the pre-synaptic cleft [13]. A diversity of dual-inhibiting molecules has developed by merging the moieties for MAOs and AChE in one compound [14]. Different research groups have identified various chemical classes bearing benzylamine [15,16], coumarin [17], oxazole, triazole [18], indole [19], quinolinone [20], isoindoline [21], and oxadiazole [22], as potent inhibitors of the targeted enzymes. Among those classes, quinoline has gained much attention as a privileged scaffold for multi-targeting enzymes (MAOs and AChE).

**Figure 1:**
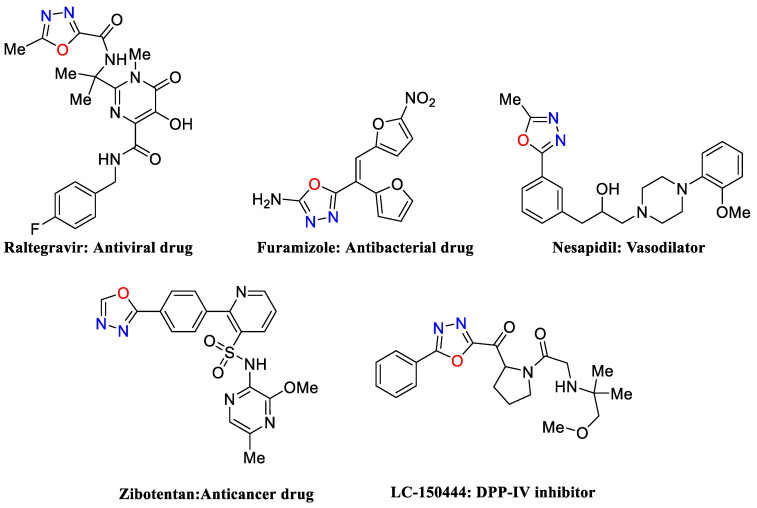
1,3,4-Oxadiazole as a pharmacophore in commercial and preclinical stage drugs.

1,3,4-Oxadiazole moiety is a heterocyclic skeleton with single O, two N, and two C atoms with compromised aromaticity and enhanced diene character [23]. Compounds belonging to this class of heterocycles have expressed a tumultuous therapeutic potential as antimicrobial [24,25,26,27], antiepileptic [28,29,30,31,32], anticancer [33,34,35,36], hypoglycemic [37,38,39,40], antiviral [41,42,43,44,45,46], and anti-inflammatory [47,48,49,50,51] agents. Meanwhile, the chemistry and biological potential of 1,3,4-oxadiazoles have been thoroughly reviewed recently [52,53,54,55,56,57]. The 1,3,4-oxadiazole moiety has become an established pharmacophore with wide availability in commercial drugs [55], including raltegravir (anti-HIV) [58], furamizole (antimicrobial) [59], nesapidil (vasodilator) [60], and zibotentan (anticancer) [61]. Similarly, LC-150444 is a 1,3,4-oxadiazole-bearing potential drug in the preclinical testing stage for the inhibition of dipeptidyl peptidase IV (DPP IV) enzyme [39]. The chemical structures of these 1,3,4-oxadiazole-bearing chemical entities are depicted in Figure 1.

Quinoline, a fused structure of benzene and pyridine at two adjacent carbon atoms, is known to possess diverse biological and pharmaceutical potential. Naturally occurring quinoline-containing compounds and synthetic derivatives of quinoline have shown enormous potential to be used as medicine or lead compounds for drug development [62]. Naturally occurring quinine found in the bark of the cinchona plant and synthetic derivatives possessing a quinoline skeleton have been fruitfully employed for the treatment of malaria [63]. Galipealongiflora tree bark contains molecules with quinolines skeleton that are used as antileishmanial agents [64]. Cryptolepineis (an indoloquinoline alkaloid), dynemicin A, and streptonigrin, are naturally occurring antitumor antibiotics [65,66]. Synthetically accessed quinoline derivatives have shown antileishmanial [67,68], DNA binding agents [68], anticancer [69,70,71,72], antimycobacterial [73,74,75], antimicrobacterial [76], anticonvulsant [77], anti-inflammatory [78,79], and cardiovascular activities [80,81].

Towards discovering new compounds for MAO and AChE enzymes inhibition, herein we are reporting the synthesis, structure elucidation, molecular modeling, and enzyme inhibition of novel S-alkyl phthalimide- and S-benzyl-oxadiazole-quinoline hybrids.

## 2. Results and Discussion

### 2.1. Chemistry

**Scheme 1:**
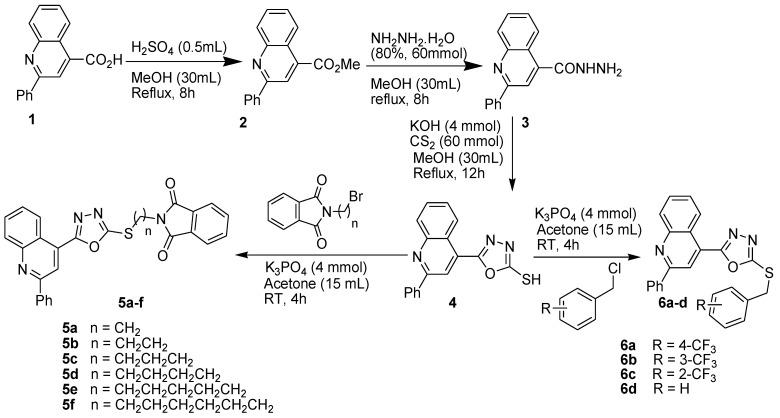
Synthesis of *S*-alkyl phthalimide- and *S*-benzyl- oxadiazole-quinoline hybrids (**5a**–**f** and **6a**–**d**).

*S*-alkyl phthalimide- and *S*-benzyl-oxadiazole-quinoline hybrids (**5a**–**f** and **6a**–**d**) were synthesized following an optimized reported procedure [82,83,84]. The synthesis was initiated by the esterification of 2-phenylquinoline-4-carboxylic acid (**1**) in methanol using a catalytic amount of sulfuric acid, giving methyl 2-phenylquinoline-4-carboxylate (**2**). Compound **2** was hydrozinolyzed in methanol with hydrazine hydrate at reflux, pursued by cyclization utilizing potassium hydroxide in methanol at reflux and then acidification at room temperature to obtain 5-(2-phenylquinolin-4-yl)-1,3,4-oxadiazole-2-thiol (**4**). The synthetic protocol is bifurcated here. On one side, 5-(2-phenylquinolin-4-yl)-1,3,4-oxadiazole-2-thiol (**4**) reacted with bromoalkyl substituted phthalimides to provide quinoline-oxadiazole-phthalimide hybrids **5a**–**f**, and on the other side, compound **4** reacted with substituted benzyl bromides to provide **6a**–**d** in excellent yields (Scheme 1).

The target molecules **5a**–**f** and **6a**–**d** were purified by recrystallization, and the structures of novel molecules were authenticated with the help of complementary analytical tools, including ^13^C-NMR, ^1^H-NMR, and FT-IR spectroscopic techniques (Figure S1). Compound **5a**’s 1H-NMR analysis revealed typical peaks in the aromatic and aliphatic areas. The singlet for the two protons at δ = 5.54 ppm represented methylene proton (*S*-CH_2_-*N*) surrounded by sulfur and nitrogen atoms. In compounds **5b**–**f**, a triplet for the methylene protons of the *S*-CH_2_ group was found between δ = 3.45 and δ = 3.26 ppm, whereas the methylene protons of *N*-CH_2_ were found between δ = 3.77 and δ = 3.56 ppm. The methylene groups between *N*-CH_2_ and *S*-CH_2_ of compounds **5c**–**f** ranged from δ = 2.19 to δ = 1.32 ppm.

The protons present in the benzylic *S*-CH_2_ group of compounds 6a–d were observed as a singlet that ranged from δ = 4.69 to δ = 4.85 ppm. The singlet for one proton on the adjacent carbon atoms of the quinoline ring was observed between δ = 8.65 and δ = 8.06 ppm. All the protons present in the aromatic rings of the quinoline and phthalimide in compounds **5a**–**f** were found in the range of δ 9.05–7.13 ppm, whereas aromatic protons present in the aromatic ring of quinoline and *S*-benzyl in compounds **6a**–**d** were observed in the range of δ 9.05–7.35 ppm.

Similarly, ^13^C NMR of compounds **5a**–**f** and **6a**–**d** showed peaks for relevant carbon atoms at appropriate positions. Carbon atoms present in the carbonyl group of phthalimide moiety in compounds **5a**–**f** were observed above δ 164 ppm. The carbon atoms O*C*N and S*C*N of the 1,3,4-oxadiazole in compounds **5a**–**f** and **6a**–**d** ring were observed between δ 164 and δ 155 ppm. All the carbon atoms present in the aromatic rings were found in the range of δ 149–118 ppm. The bridging carbon atom (S*C*N) present in **5a** was observed at δ 39.13 ppm. The two bridging carbon atoms (S*CC*N) present between *S* and *N* atoms in compound **5b** were observed at δ 37.13 and 30.14 ppm. The carbon atoms linked to the *N* atom were found in the range of δ 38–35 ppm. The carbon atoms linked to *S* atom were found in the range of δ 32–30 ppm. Other carbon atoms of the alkyl chain between *N* and *S* atoms in **5c**–**f** were observed below δ 30 ppm. The benzylic carbon of compounds **6a**–**d** was observed between δ 35.98 and δ 33.51 ppm.

### 2.2. Inhibitory Activity and SAR

In the synthesized compounds, the oxadiazole ring was substituted with quinoline on the left side. Besides, the oxadiazole ring was replaced with an isoindoline on the right side. The position of different functional groups on the basic pharmacophore ((2-phenylquinolin-4-yl)-1,3,4-oxadiazol-2-yl)) ring was studied to get specific information about the identification and selectivity of compounds to inhibit MAOs and AChE enzymes. Two series of compounds (**5a**–**f** and **6a**–**d**) showed activity in low μM to nM ranges on the inhibition of MAOs and AChE enzymes (Table 1). Clorgyline and deprenyl were utilized as reference ligands for MAO-A and MAO-B, respectively. At the same time, donepezil was utilized as a positive AChE control. Compounds **5a** and **5f** showed activity in submicromolar ranges against MAO-A and MAO-B (IC_50_ = 0.91 ± 0.15 and 0.84 ± 0.06 μM, respectively). For AChE enzyme inhibition, compound **5c** demonstrated the most active compound (IC_50_ = 1.02 ± 0.65 μM).

As the length of the carbon chain increases between the oxadiazole ring and isoindoline, activity toward MAO-A decreases as **5b**, **5c**, **5d**, **5e**, and **5f** with values of 1.81 ± 0.38, 3.31 ± 0.80, 3.18 ± 1.23, 4.14 ± 0.35, and 4.88 ± 1.75 μM, respectively. In addition, when the isoindoline ring was replaced by a phenyl ring with different substitution CF_3_ at the ortho, para, and meta positions (**6c**, **6b**, and **6a**), the activity toward MAO-A increased by 6.81 ± 2.65, 1.51 ± 0.52, and 1.02 ± 0.92 μM, respectively.

By increasing the length of the carbon chain, activity toward MAO-B decreases as **5a**, **5b**, **5c**, **5d**, and **5e** with values of 1.59 ± 1.66, 2.61 ± 2.48, 3.39 ± 0.42, 3.76 ± 1.04, and 3.84 ± 0.91 μM, respectively. While in the case of long carbon chain **5f**, inhibition toward MAO-B was 0.84 ± 0.06 μM. Besides, when the isoindoline ring is replaced by a phenyl ring with different CF_3_ substitution at the ortho, para, and meta position (**6a**–**c**), the activity toward MAO-B increases with values of 5.59 ± 3.22, 3.71 ± 2.88, and 2.71 ± 0.88 μM, respectively.

Toward AChE inhibition, as the length of the carbon chain increases, the inhibition activity decreases as **5a**, **5c**, **5e**, **5b**, **5d**, and **5f** with IC_50_ values of 1.40 ± 0.45, 1.02 ± 0.65, 1.29 ± 0.75, 2.55 ± 0.96, 2.38 ± 0.92, and 3.32 ± 0.45 μM, respectively. While, by the replacing of the isoindoline ring by a phenyl ring with different substitution CF_3_ at the ortho, meta, and para position (**6c**, **6b**, and **6a**, respectively), the activity toward AChE increases 4.38± 1.45, 3.54 ± 1.05, and 3.23 ± 0.95 μM, respectively.

**Figure 2:**
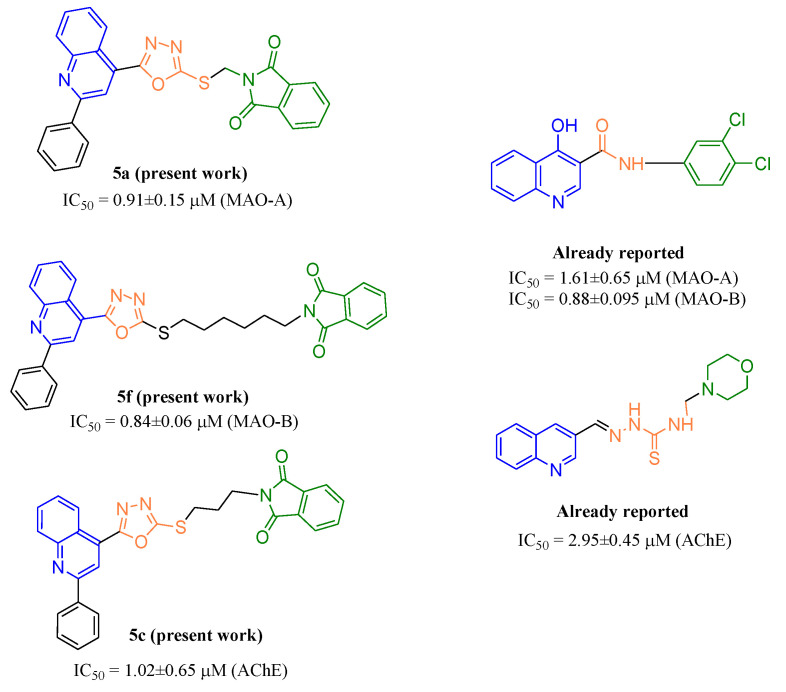
Potent compounds on MAO-A, MAO-B, and AChE enzymes.

The correlation of newly synthesized (2-phenylquinolin-4-yl)-1,3,4-oxadiazol isoindoline-1,3-dione) with previously known MAO and AChE inhibitors was investigated. It seemed that the synthesized compounds showed higher inhibition activity toward targeted enzymes compared to the reported ones (Figure 2) [85,86]. Moreover, the synthesized compounds showed dual and multi-targeted inhibition. 

### 2.3. Kinetic Studies

**Figure 3:**
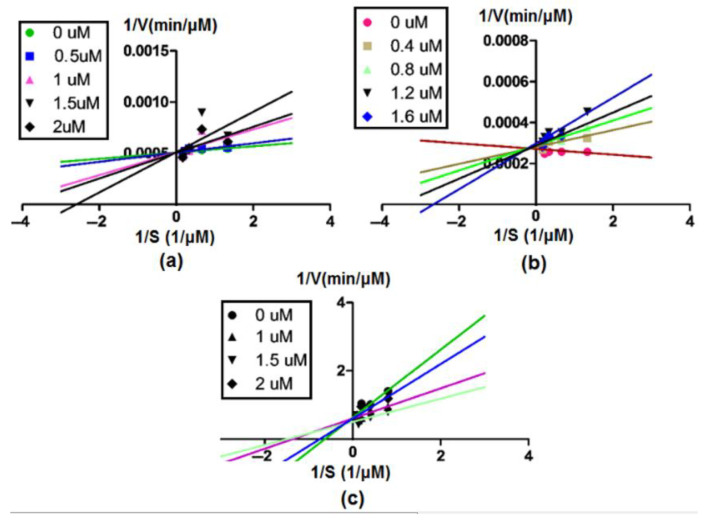
The double reciprocal plot of (**a**) MAO-A, (**b**) MAO-B, and (**c**) AChE activity in the presence and absence of different concentrations of the most promising compounds (**5a**, **5f**, and **5c**).

To determine the method of targeting MAO-A, MAO-B, and AChE enzymes, kinetic experiments for the most potent molecules (**5a**, **5f**, and **5c**) were carried out (Figure 3). Different test chemicals and substrate concentrations were utilized in the kinetic experiments. Lineweaver-Burk plots were employed to determine the kind of inhibition, and they were used to track how the inhibitor affected *K*_m_ and *V*_max_ by plotting the reciprocal reaction rate against the reciprocal substrate concentrations. As depicted in Figure 3, the investigated molecules displayed a pure competitive type of inhibition as the *V*_max_ of enzymes was unaffected by differing doses of the test compounds while the *K*_m_ increased.

### 2.4. Docking Studies

**Figure 4:**
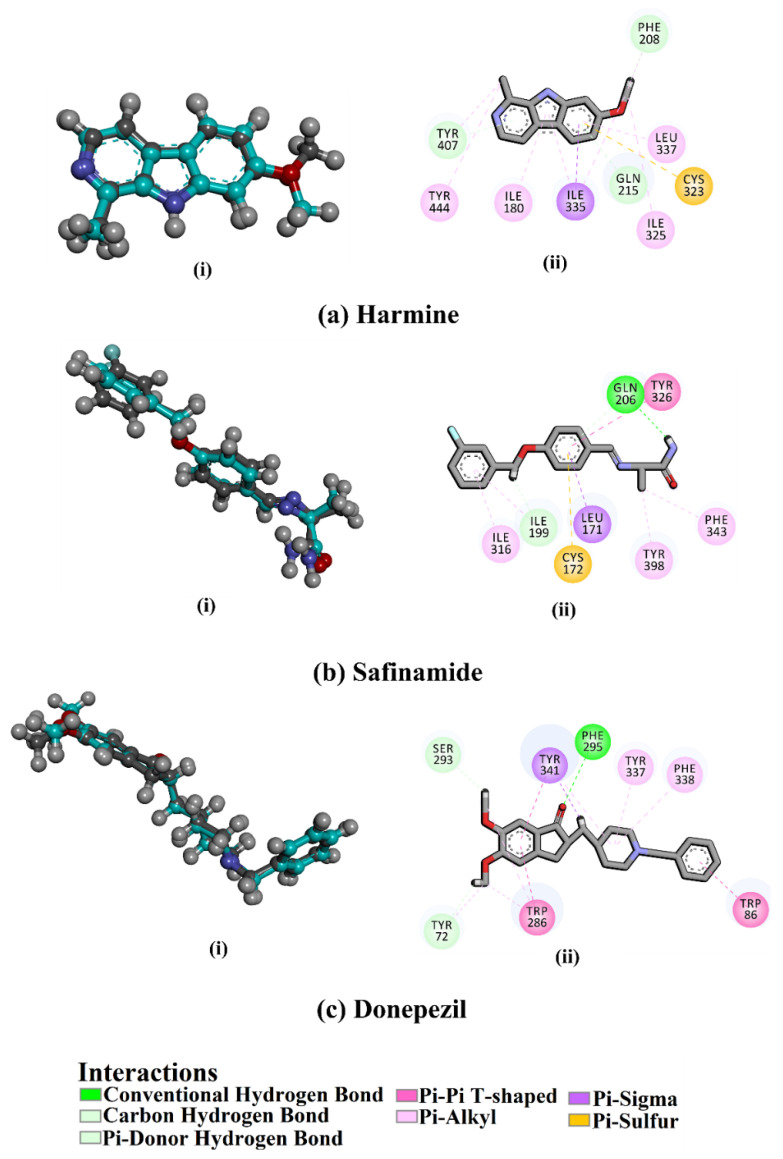
(**i**) 3D molecular interaction of the native structure (in cyan) and the anticipated docking pose (in gray) and (**ii**) 2D molecular interaction of the anticipated docking pose of (**a**) harmine with MAO-A, (**b**) safinamide with MAO-B, and (**c**) donepezil with AChE.

To investigate the docking pose of the most promising molecules with the binding pocket of the targeted enzymes, molecular docking predictions were executed. The assessment of the AutoDock4.2.6 software with the utilized settings was first carried out in accordance with the accessible experimental data. The co-crystallized ligands –namely, harmine, safinamide, and donepezil– with the MAO-A, MAO-B, and AChE were re-docked and compared to the experimentally resolved structures (PDB codes: 2Z5X, 2V5Z, and 4EY7, respectively) (Figure 4). As depicted in Figure 4, the portended docking poses resembled the native structures, having 0.23, 0.27, and 0.24 Å RMSD in relation to the co-crystallized conformations of harmine, safinamide, and donepezil, respectively (Figure 4). Summing up, the employed docking protocol would be utilized to predict the correct docking pose of inhibitors with the targeted enzymes.

**Figure 5:**
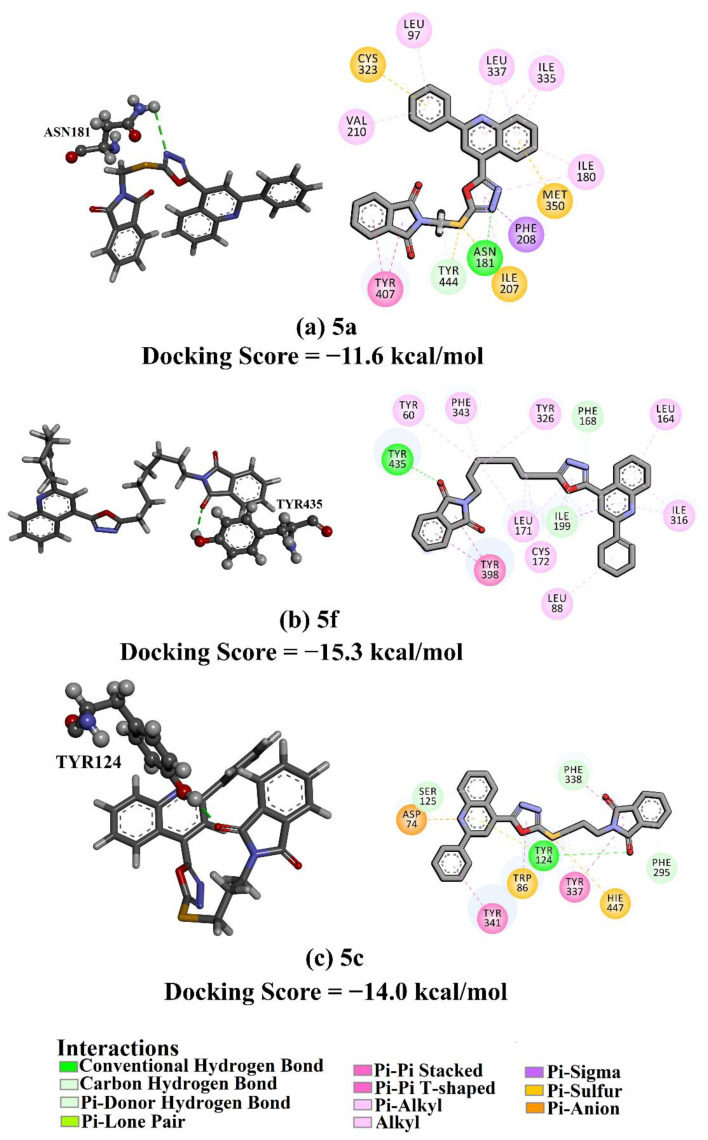
Predicted docking scores (in kcal/mol) and docking poses of (**a**) **5a** with MAO-A, (**b**) **5f** with MAO-B, and (**c**) **5c** with AChE.

Utilizing the docking protocol, the binding scores and modes of **5a**, **5f,** and **5c** with MAO-A, MAO-B, and AChE enzymes, respectively, were anticipated (Figure 5). As illustrated in Figure 5, the investigated compounds demonstrated good inhibition affinity toward the inspected enzymes, with docking scores of –11.6, –15.3, and –14.0 kcal/mol with MAO-A, MAO-B, and AChE, respectively. The monitored possibility of inspected compounds towards MAO-A, MAO-B, and AChE might be ascribed to their capacity to exhibit numerous H-bonds, hydrophobic, vdW, and π-based interactions with the substantial residues within the binding pockets of these enzymes.

When the results were compared with the co-crystallized conformations of harmine, safinamide, and donepezil, many common interactions were observed (Figure 5). The presence of a quinoline ring formed the π-alkyl interactions with ILE180, LEU337, ILE335, LEU164, and ILE316 amino acids (Figure 5). Similarly, the 1,3,4-oxadiazol and isoindoline-,3-dione ring exhibited hydrogen bond, π-π stacked, and π-amide interactions with ASN141, TYR435, TYR124, TYR405, TYR398, TYR337, and TYR341 (Figure 5). 

### 2.5. Drug-Like and ADMET Characteristics

The drug-like and ADMET features of the potent molecules were computed and compared with clorgyline, deprenyl, and donepezil as controls (Table 2). A compound’s hydrophilicity is indicated by Log P; if the value is negative, the compound is hydrophilic. All of the compounds in Table 2 are lipophilic. The total number of OH and NH atoms equals the nHBD, while the total number of N and O atoms resembles the nHBA. As listed in Table 2, the nHBA and nHBD are in the optimal ranges (nHBD < 5 and nHBA < 10). The ideal lipophilicity for BBB penetration for medications having CNS activity is a Log D ≤ 2. A Log D of more than 4 is considered as not suitable for a CNS medication. In vivo intestinal medication absorption is demonstrated by CaCo-2 permeability values. The ideal range of values should be more than −5.15 Log unit. All compounds showed normal values of CaCo-2 permeability. The higher HIA value, the higher intestinal absorption will be. All studied molecules pointed out an appropriate HIA value compared to the controls. Because of their lipophilic nature, all of our compounds can cross BBB. In metabolism and excretion, all investigated compounds were inhibitors of CYP2C19 (Table 2). A drug’s bodily clearance rate is categorized as high (>15), moderate (5–15), or low (<5). All the newly synthesized compounds had a low clearance rate. The AMES toxicity was evaluated. All compounds manifested low toxicity. Therefore, it can be suggested that all potent derivatives have a good ADMET profile compared to the controls. Further investigation is required to improve the toxicity profile of compounds.

### 2.6. Density Function Theory (DFT) Calculations

**Figure 6:**
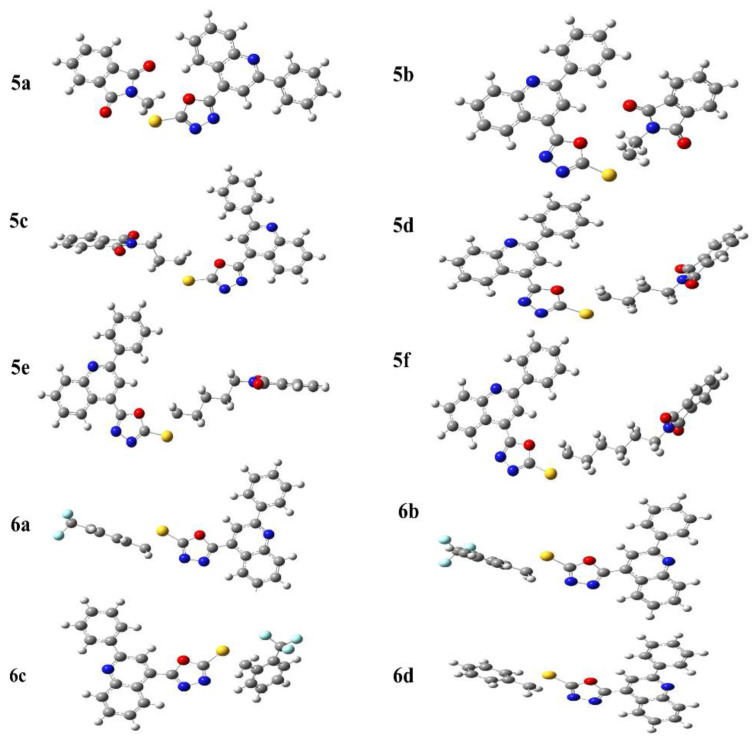
Optimized structures of compounds **5a**–**f** and **6a**–**d**.

**Figure 7:**
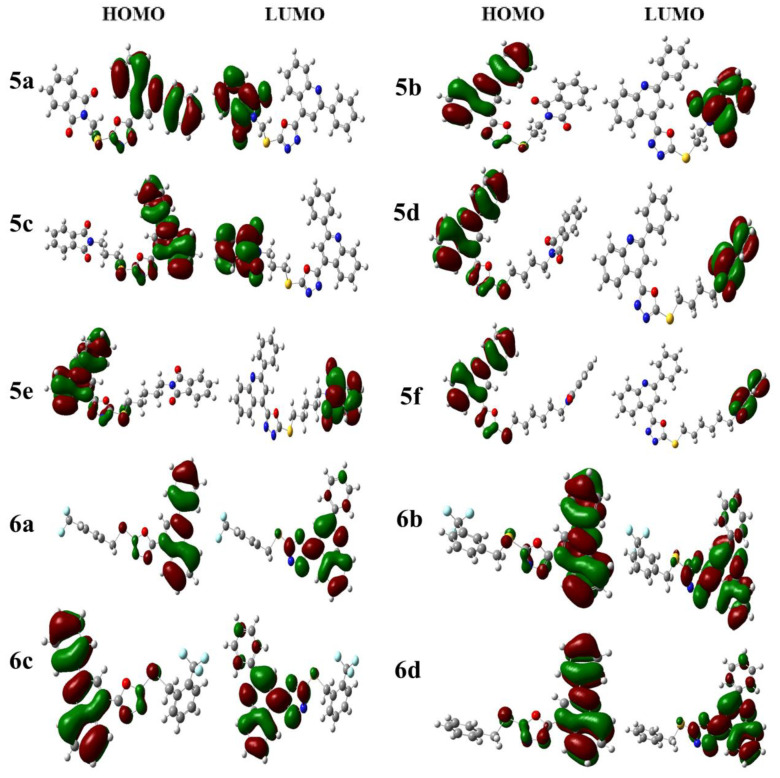
Diagrams of HOMO and LUMO distributions of compounds **5a**–**f** and **6a**–**d**.

Further insight into the features of the investigated compounds was traced using a plethora of quantum mechanical calculations. In the first place, geometrical optimization was executed at the B3LYP/6-31G level of theory, and the obtained structures are illustrated in Figure 6. Afterwards, frequency computations were exerted, outlining that the optimized structures were true minima with no observable imaginary frequency values. Upon the optimized compounds, single-point energy and Frontier molecular orbitals (FMO) calculations were performed, and the global reactivity parameters were evaluated. Diagrams of HOMO and LUMO distributions are displayed in Figure 7. Table 3 enrolls energies of the optimized compounds (*E*_opt_), the highest occupied molecular orbital (*E*_HOMO_), and the lowest unoccupied molecular orbital (*E*_LUMO_), along with the energy gap (*E*_gap_), global hardness (*ƞ*), global softness (*σ*), polarizability (*α*), and dipole moment (*μ*) of the studied compounds.

In the context of FMO theory, the molecule with less and high negative values of *E*_HOMO_ and *E*_LUMO_, respectively, had superior nucleophilic nature. For ∆*E*_gap_, less positive values ensured the noticeable ability of the inspected compound to donate electrons. 

The most reactive compound would be the one that has the smallest energy gap. The most kinetically stable compound showed a more favorable energy gap compared to the others. As evident in Figure 7, HOMO and LUMO distributions were noticed with lower and higher concentrations, respectively, around the phthalimide group in **5a**–**f** compounds. While both distributions were found over the 5-(2-phenylquinolin-4-yl)-1,3,4-oxadiazole-2-thiol (**4**) in the **6a**–**d** compounds.

According to data listed in Table 3, compound 5a would be highly reactive with favorable nucleophilic nature. This observation could be ascribed to the least negative *E*_HOMO_ (–0.221 eV), highest negative *E*_LUMO_ (–0.112 eV), and least positive *E*_gap_ (0.108 eV) values. In comparison, the reversed affirmations were generally noticed in the case of compound **6d**, which in turn was addressed as the most kinetically stable compound. The computed values of the global reactivity parameters ensured the FMO affirmations.

## 3. Experimental

### 3.1. Materials and Apparatus

All commercially obtained chemicals and reagents were employed to accomplish the targeted synthesis. Solvents of analytical grade were employed as provided. Uncorrected melting points were evaluated in open capillaries by a Gallenkamp melting point instrument (MP-D). Thin-layer chromatography was employed to analyze all the reactions. It was executed on Merck pre-coated plates (silica gel 60 F254, 0.25 mm), and was used to visualize the reactions utilizing fluorescence quenching under UV light (254 nm). A Bruker AV-300 spectrometer was applied to measure the ^1^H-NMR and ^13^C-NMR spectra (300 MHz). ATR was used to record IR spectra on a Shimadzu Fourier Transform Infrared spectrophotometer model (Attenuated Total Reflectance).

### 3.2. General Procedure

A modified multistep synthetic procedure was followed to access **5a**–**f** and **6a**–**d**.

In a typical experimental procedure, 2-phenylquinoline-4-carboxylic acid (**1**) (30 mmol) was esterified in 20 mL of methanol using catalytic amounts of sulfuric acid (0.5 mL) at reflux temperature for 4 h. The mixture was neutralized with 50 mL of saturated NaHCO_3_ solution and extracted three times with 30 mL of ethyl acetate. The organic phase was filtered after being dried over anhydrous sodium sulfate. The solvent was then extracted to get crude methyl 2-phenylquinoline-4-carboxylate in quantitative yield.

Methyl 2-phenylquinoline-4-carboxylate (**2**) (25 mmol) was dissolved in 30 mL of CH_3_OH, and NH_2_NH_2_.H_2_O (80%, 0.06 mol) was inserted dropwise. The reaction mixture was cooled to room temperature and then poured into ice-cold water after 8 h of refluxing. We precipitated, filtered, dried, and recrystallized 2-phenylquinoline-4-carbohydrazide (**3**) from methanol.

A solution of 2-phenylquinoline-4-carbohydrazide (**3**) (20 mmol) in 10 mL of methanol and 3 equivalent KOH dissolved in 30 mL of methanol was inserted. After 10 min, carbon disulfide (30 mmol) was slowly inserted, and the whole reaction mixture was subjected to reflux for 12 h. The reaction mixture was concentrated, brought to room temperature, and then added to ice water. With dilute HCl, the pH of the solution was brought down to 2. Warm water was used to wash the precipitated 5-(2-phenylquinolin-4-yl)-1,3,4-oxadiazole-2-thiol (**4**) before it was recrystallized from methanol. 

Acetone was used to dissolve 5-(2-phenylquinolin-4-yl)-1,3,4-oxadiazole-2-thiol (**4**) (3 mmol), and potassium carbonate (4 mmol) was added. N-bromoalkylphthalimide (4 mmol) was added to the reaction mixture after it had been agitated at room temperature for 10 min. The reaction mixture was then mixed once more at room temperature for 6 h. To obtain pure **5a**–**f**, the crude was recrystallized from methanol after the solvent was removed.

Acetone was utilized to dissolve 5-(2-Phenylquinolin-4-yl)-1,3,4-oxadiazole-2-thiol (**4**) (3 mmol), and potassium carbonate (4 mmol) was added. Substituted benzyl bromide (4mmol) was added to the reaction mixture after it had been agitated at room temperature for 10 min. The reaction mixture was then mixed once more at room temperature for 6 h. To get pure **6a**–**d**, the crude was recrystallized from methanol.

#### 3.2.1. 2-((5-(2-Phenylquinolin-4-yl)-1,3,4-oxadiazol-2-ylthio)methyl)isoindoline-1,3-dione (**5a**)


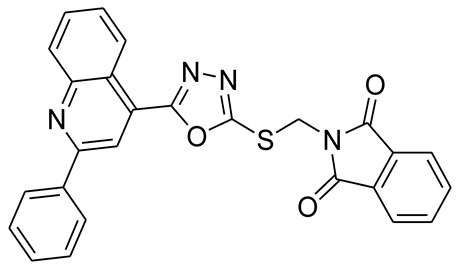


Off white solid; yield: 66%, R_f_: 0.72 (Chloroform: acetone, 9:1); mp:178–180 °C; 1H-NMR (300 MHz, DMSO-d6): δ (ppm); 9.05 (d, *J* = 9 Hz, 1H), 8.65 (s, 1H), 8.39 (d, *J* = 9 Hz, 2H), 8.20 (d, *J* = 9 Hz, 1H), 7.84 (m, 6H), 7.62 (m, 3H), 5.54 (s, 2H);13C NMR (75 MHz, DMSO-d6); 166.98, 164.88, 163.09, 156.34, 148.74, 138.12, 135.40, 131.87, 131.24, 130.69, 130.53, 129.49, 128.95, 128.55, 127.26, 123.95, 122.36, 118.91 39.13; FT-IR υ (cm^−1^): 3006 (C-H, SP2), 2930 (C-H, SP3), 1717 (C = O), 1614, 1595 (C = N); HR/MS (EI): *m/z* calculated for C26H16N4O3S: 464.0943; found 464.0947. 

#### 3.2.2. 2-(2-(5-(2-Phenylquinolin-4-yl)-1,3,4-oxadiazol-2-ylthio)ethyl)isoindoline-1,3-dione (**5b**)


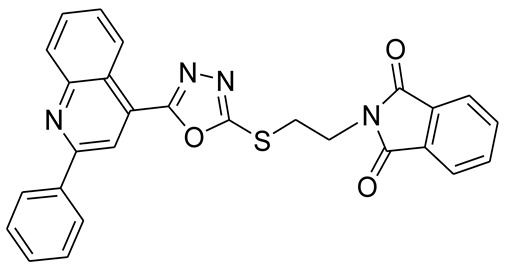


Off white solid; yield: 83%, R_f_: 0.76 (Chloroform: acetone, 9:1); mp:198–200 °C; ^1^H-NMR (300 MHz, DMSO-d_6_): δ (ppm); 8.46 (d, *J* = 9 Hz, 1H), 8.06 (s, 1H), 7.92 (d, *J* = 6 Hz, 2H), 7.72 (d, *J* = 9 Hz, 1H), 7.45 (t, *J* = 15 Hz, 1H), 7.26 (m, 5H), 7.139d, *J* = 6 Hz, 3H), 3.66 (t, *J* = 12 Hz, 2H), 3.26 (t, *J* = 12 Hz, 2H); ^13^C NMR (75 MHz, DMSO-d_6_); 167.65, 164.63, 163.46, 155.83, 148.27, 137.66, 134.39, 131.38, 130.73, 130.17, 130.03, 128.99, 128.40, 127.90, 127.38, 125.52, 123.00, 121.81, 118.17.37.13, 30.54; FT-IR υ (cm^−1^): 3055 (C-H, SP^2^), 2942 (C-H, SP^3^), 1714 (C = O), 1594, 1525 (C = N); HR/MS (EI): *m/z* calculated for C_27_H_18_N_4_O_3_S: 478.5218; found 478.5220.

#### 3.2.3. 2-(3-(5-(2-Phenylquinolin-4-yl)-1,3,4-oxadiazol-2-ylthio)propyl)isoindoline-1,3-dione (**5c**)


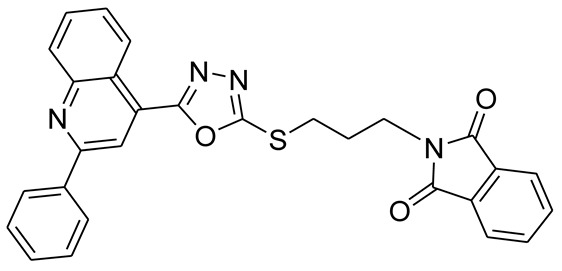


Off white solid; yield: 80%, R_f_: 0.77 (Chloroform: acetone, 9:1); mp: 154–158 °C; ^1^H-NMR (300 MHz, DMSO-d_6_): δ (ppm); 8.98 (d, *J* = 9 Hz, 1H), 8.51(s, 1H), 8.31 (d, *J* = Hz, 2H), 8.18 (d, *J* = 9 Hz, 1H), 7.81 (m, 6H), 7.55 (d, *J* = 6 HZ, 3H), 3.77 (t, *J* = 15 Hz, 2H), 3.45 (t, *J* = 12 Hz, 2H), 2.19 (t, *J* = 12 Hz, 2H); ^13^C NMR (75 MHz, DMSO-d_6_); 168.56, 165.50, 164.07, 146.28, 148.75, 138.10, 134.76, 132.19, 131.18, 130.60, 130.49, 129.44, 128.83, 128.64, 127.78, 126.02, 123.44, 122.38, 118.66, 36.64, 30.14, 28.; FT-IR υ (cm^−1^): 3047 (C-H, SP^2^), 2943 (C-H, SP^3^), 1702 (C = O), 1599, 1536 (C = N); HR/MS (EI): *m/z* calculated for C_29_H_22_N_4_O_3_S: 492.5484; found 492.5487.

#### 3.2.4. 2-(4-(5-(2-Phenylquinolin-4-yl)-1,3,4-oxadiazol-2-ylthio)butyl)isoindoline-1,3-dione (**5d**)


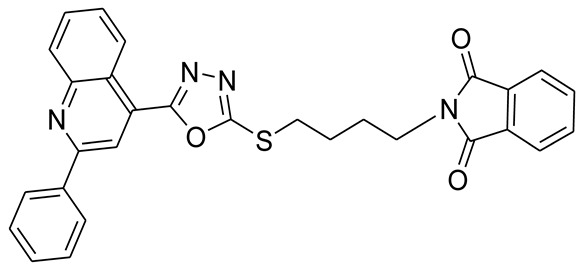


White solid; yield: 86%, R_f_: 0.76 (Chloroform: acetone, 9:1); mp: 121–125 °C; ^1^H-NMR (300 MHz, DMSO-d_6_): δ (ppm); 8.97 (d, *J* = 9 Hz, 1H), 8.48 (s, 1H), 8.30 (d, *J* = 6 Hz, 2H), 8.17 (d, *J* = 9 Hz, 1H), 7.89 (t, *J* = 15 Hz, 1H), 7.77 (m, 5H), 7.56 (d, *J* = 9 Hz, 3H), 3.64 (t, *J* = 9 Hz, 2H), 3.42 (t, *J* = 9 Hz, 2H), 1.78 (m, 4H); ^13^C NMR (75 MHz, DMSO-d_6_); 168.43, 165.52, 164.00, 156.26, 148.75, 138.11, 134.75, 132.02, 131.16, 130.58, 130.48, 129.44, 128.81, 128.62, 127.75, 126.04, 123.40, 122.36, 118.62, 37.31, 32.13, 27.33, 26.82; FT-IR υ (cm^−1^): 3040 (C-H, SP^2^), 2943 (C-H, SP^3^), 1707 (C = O), 1598, 1549 (C = N); HR/MS (EI): *m/z* calculated for C_28_H_20_N_4_O_3_S: 506.1413; found 506.1416.

#### 3.2.5. 2-(5-(5-(2-Phenylquinolin-4-yl)-1,3,4-oxadiazol-2-ylthio)pentyl)isoindoline-1,3-dione (**5e**)


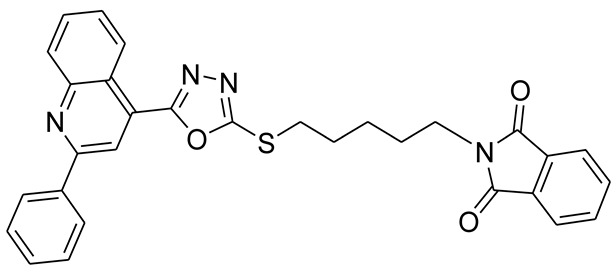


White solid; yield 74%, R_f_: 0.67 (Chloroform: acetone, 9:1); mp: 146–150 °C; ^1^H-NMR (30 0MHz, DMSO-d_6_): δ (ppm); 8.99 (d, *J* = 6 Hz, 1H), 8.49 (s, 1H), 8.29 (d, *J* = 6 Hz, 2H), 8.17 (j = 9 Hz, 1H), 7.88 (t, *J* = 15 Hz, 1H), 7.75 (m, 5H), 3.58 (t, *J* = 15 Hz, 2H), 3.38 (t, *J* = 18 Hz, 2H), 1.88 (pnt, *J* = 15, 9 Hz, 2H), 1.64 (m, 2H), 1.47 (t, *J* = 15 Hz, 2H);^13^C NMR (75 MHz, DMSO-d_6_); 168.39, 165.59, 163.98, 156.27, 148.76, 138.11, 134.77, 132.00, 131.15, 130.57, 130.48, 129.43, 128.79, 128.67, 127.73, 126.04, 123.40, 122.38, 118.62, 37.64, 32.35, 28.98, 27.88, 25.64; FT-IR υ (cm^−1^): 3060 (C-H, SP^2^), 2933 (C-H, SP^3^), 1713 (C = O), 1597, 1534 (C = N); HR/MS (EI): *m/z* calculated for C_30_H_24_N_4_O_3_S: 520.1569; found 520.1572.

#### 3.2.6. 2-(6-(5-(2-Phenylquinolin-4-yl)-1,3,4-oxadiazol-2-ylthio)hexyl)isoindoline-1,3-dione (**5f**)


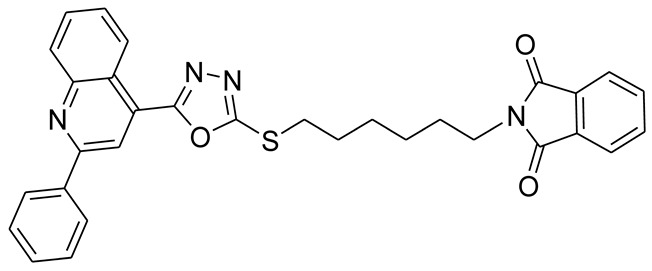


White solid; yield 73%, R_f_: 0.85 (Chloroform: acetone, 9:1); mp: 132–135 °C; ^1^H-NMR (300 MHz, DMSO-d_6_): δ (ppm); 8.97(d, *J* = 9 Hz, 1H), 8.48 (s, 1H), 8.29 (t, *J* = 9 Hz, 2H), 8.16 (d, *J* = 9 Hz, 1H), 7.88 (t, *J* = 12 Hz, 1H), 7.77 (m, 5H), 7.56 (m, 3H), 3.56 (t, *J* = 18 Hz, 2H), 3.37 (t, *J* = 15 Hz, 2H), 1.81 (pnt, *J* = 12, 6 Hz, 2H), 1.59 (pnt, *J* = 15, 9 Hz, 2H), 1.46 (pnt, *J* = 18, 9 Hz, 2H), 1.32 (pnt, *J* = 15, 6 Hz, 2H); ^13^C NMR (75 MHz, DMSO-d_6_); 168.38, 165.68, 163.98, 156.28, 148.76, 138.11, 134.77, 132.02, 131.17, 130.60, 130.49, 129.44, 128.82, 128.69, 127.74, 126.03, 123.40, 122.39, 118.65, 37.70, 32.43, 29.26, 28.22, 27.88, 26.12; FT-IR υ (cm^−1^): 30,457 (C-H, SP^2^), 2933 (C-H, SP^3^), 1717 (C = O), 1596, 1536 (C = N); HR/MS (EI): *m/z* calculated for C_31_H_26_N_4_O_3_S: 534.1726; found 534.1728.

#### 3.2.7. 4-(5-(4-(Trifluoromethyl)benzylthio)-1,3,4-oxadiazol-2-yl)-2-phenylquinoline (**6a**)


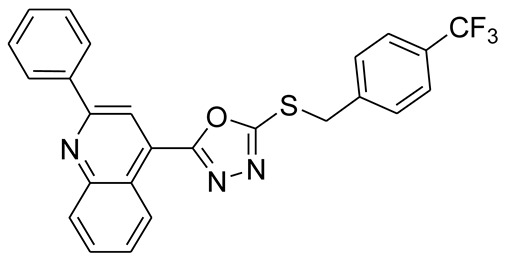


White solid; yield: 74%, R_f_: 0.84 (n-hexane: ethylacetate; 6:4); mp: 144–145 °C; ^1^H-NMR (300 MHz, CDCl_3_): δ (ppm); 9.14 (d, *J* = 9 Hz, 1H), 8.38 (s, 1H), 8.23 (m, 3H), 7.84 (m, 1H), 7.70 (m, 5H), 7.55 (m, 3H), 4.65 (s, 2H); ^13^C NMR (75 MHz, CDCl_3_); 164.66, 164.53, 156.74, 149.19, 139.71, 138.60, 130.66, 130.52, 130.47,130.22, 129.92, 129.61, 129.02, 128.27, 128.18, 127.46, 125.89, 125.85, 125.80, 122.44, 122.11, 118.32, 35.98; FT-IR υ (cm^−1^): 3064 (C-H, SP^2^), 2950 (C-H, SP^3^), 1599 (C = N), 1325 (C-S); HR/MS (EI): *m/z* calculated for C_25_H_16_F_3_N_3_OS: 463.0966; found 463.0969.

#### 3.2.8. 4-(5-(3-(Trifluoromethyl)benzylthio)-1,3,4-oxadiazol-2-yl)-2-phenylquinoline (**6b**)


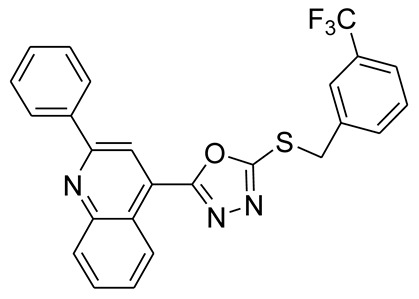


White solid; yield: 76%, R_f_: 0.64 (n-hexane: ethylacetate; 6:4); mp: 134–135 °C; ^1^H-NMR (300 MHz, CDCl_3_): δ (ppm); 8.97 (d, *J* = 9 Hz, 1H), 8.48 (s, 1H), 8.29 (dd, J 9, 6 Hz, 2H), 8.17 (d, *J* = 9 Hz, 1H), 7.95 (s, 1H), 7.86 (dd, *J* = 15, 6 Hz, 2H), 7.74 (m, 1H), 7.59 (m, 5H), 4.78 (s, 2H); ^13^C NMR (75 MHz, CDCl_3_; 164.84, 164.35, 156.25, 148.74, 139.55, 138.99, 138.06, 133.77, 131.17, 130.61, 130.49, 130.12, 129.88, 129.42, 128.84, 128.50, 127.73, 126.32, 126.20, 125.98, 124.97, 124.92, 122.71, 122.31.118.65, 35.48; FT-IR υ (cm^−1^): 3054 (C-H, SP^2^), 2951 (C-H, SP^3^), 1595 (C = N), 1327 (C-S); HR/MS (EI): *m/z* calculated for C_25_H_16_F_3_N_3_OS: 463.0966; found 463.0968.

#### 3.2.9. 4-(5-(2-(Trifluoromethyl)benzylthio)-1,3,4-oxadiazol-2-yl)-2-phenylquinoline (**6c**)


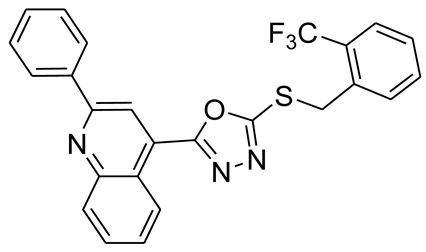


White solid; yield: 76%, R_f_: 0.81 (n-hexane: ethylacetate; 6:4); mp: 122–123 °C; ^1^H-NMR (300 MHz, CDCl_3_): δ (ppm); 8.98 (d, *J* = 9 Hz, 1H), 8.45 (s, 1H), 8.28 (m, 2H), 8.17 (d, *J* = 6 Hz, 1H), 7.89 (m, 2H), 7.73 (m, 3H), 7.56 (m, 4H), 4.85 (s, 2H); ^13^C NMR (75 MHz, CDCl_3_; 164.45, 164.42, 156.23, 148.75, 138.05, 134.69, 133.60, 132.52, 131.20, 130.63, 130.50, 129.44, 129.29, 128.87, 128.50, 127.88, 128.50, 127.88, 127.70, 127.48, 126.93, 126.86, 126.57, 125.96, 122.33, 33.51; FT-IR υ (cm^−1^): 3065 (C-H, SP^2^), 2950 (C-H, SP^3^), 1599 (C = N), 1346 (C-S); HR/MS (EI): *m/z* calculated for C_25_H_16_F_3_N_3_OS: 463.0966; found 463.0970.

#### 3.2.10. 4-(5-(Benzylthio)-1,3,4-oxadiazol-2-yl)-2-phenylquinoline (**6d**)


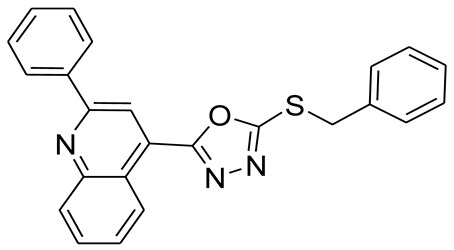


White solid; yield: 74%, R_f_: 0.85 (n-hexane: ethylacetate; 6:4); mp: 139–140 °C; ^1^H-NMR (300 MHz, DMS0-d_6_): δ (ppm); 8.98 (d, *J* = 9 Hz, 1H), 8.46 (s, 1H), 8.29 (d, *J* = 6 Hz, 2H), 8.16 (d, *J* = 6 Hz, 1H), 7.88 (t, *J* = 15 Hz, 1H), 7.75 (t, *J* = 15 Hz, 1H), 7.58 (m, 5H), 7.35 (m, 3H), 4.69 (s, 2H);^13^C NMR (75 MHz, DMS0-d_6_; 165.09, 164.21, 156.26, 148.75, 138.08, 137.04, 131.18, 130.63, 130.49, 129.59, 129.45, 129.10, 128.86, 128.52, 128.30, 127.75, 126.01, 122.32, 118.63, 36.28; FT-IR υ (cm^−1^): 3062 (C-H, SP2), 2950 (C-H, SP3), 1595 (C = N), 1336 (C-S); HR/MS (EI): *m/z* calculated for C_24_H_17_F_3_N_3_OS: 395.1092; found 395.1095.

### 3.3. Inhibition Assay Protocol

#### 3.3.1. Monoamine Oxidase

According to the previously reported protocol, the synthesized compounds’ action on the monoamine oxidase (MAO-A and MAO-B) enzymes was tested. The enzyme was produced just 15 to 20 min before, at a cold room temperature. Accordingly, clorgyline (60 nM) or deprenyl (300 nM) were used to permanently inhibit MAO-A and MAO-B activity. White 96-well plates were utilized for the test. The assay volume was 100 μL, having 60 μL buffer (pH 7.4) and 10 μL test compound (0.1 mM, 10% DMSO), followed by adding enzyme 10 μL (26 μg of protein for MAO-A and 5.0 μg for MAO-B). For MAO-A and MAO-B, the mixture was incubated for 20 and 15 min, respectively. The mixture was then given 10 μL of the substrate and 10 μL of newly prepared Amplex red. Accordingly, the final concentrations of clorgyline and deprenyl were utilized to calculate the activities of non-MAO-A and MAO-B. Using a fluorescence plate reader (BMG Labtech GmbH, orten berg Germany), the change in fluorescence was identified. The compounds that showed inhibition of either MAO-A or MAO-B activity of >50% underwent additional testing to determine their IC_50_ values. The non-linear curve fitting tool PRISM 5.0 (GraphPad, San Diego, CA, USA) was employed to compute IC_50_ values.

#### 3.3.2. Acetylcholinesterase

All substances were put through a little modified version of Ellman’s test to gauge the effectiveness of their ability to inhibit acetylcholinesterase (AChE). Released thiocholine reacts with chromogenic reagent 5,5-dithio-bis (2-nitrobenzoic) acid to produce a colorful product (DTNB). At a concentration of 2.5 units/mL, the enzyme solutions were prepared. The assay volume was 100 μL, having 60 μL buffer and 10 μL test compound (0.1 mM, 10% DMSO), pursued by inserting enzyme 10 μL (0.04 U/well). The mixture was incubated for 10 min. 10 μL of the substrate and 10 μL of DTNB were added to the mixture. After 30 min, the production of the yellow anion was recorded at 405 nm.

### 3.4. Molecular Docking and ADMET Characteristics

#### 3.4.1. Enzyme Preparation

The X-ray structures of MAO-A (PDB code: 2Z5X [87]), MAO-B (PDB code: 2V5Z [88]), and AChE (PDB code: 4EY7 [89]) were opted as templates for all docking predictions. The enzymes were prepared by excluding all ions, heteroatoms, water molecules, and ligands. The Modeller software was applied to create all missing residues [90]. The investigated enzymes’ protonation states were examined using the H++ website [91]. All missing hydrogen atoms were inserted.

#### 3.4.2. Inhibitor Preparation

The 3D molecular structures of the inspected molecules **5a**–**f** and **6a**–**d** were manually constructed. Before any computation, all investigated compounds were firstly minimized using the MMFF94S force field implemented inside the SZYBKI software [92,93]. The charges of all investigated compounds were computed utilizing the Gasteiger-Marsili method [94].

#### 3.4.3. Docking Calculations

The AutoDock4.2.6 software was employed to execute all docking computations [95]. Based on the AutoDock protocol, the pdbqt file for the examined enzymes was generated utilizing the MGL (molecular graphics laboratory) tools 1.5.7 [96]. In general, the docking parameters of the AutoDock4.2.6 software were maintained at their default settings. The GA (number of genetic algorithms) run variables was 250. The eval (maximum number of energy evaluations) was 25,000,000. The grid box size was 50 Å × 50 Å × 50 Å. For all docking engines, the cartesian coordinates of the grid center were located at the center of the binding pockets of the investigated enzymes. The AutoGrid4.2.6 program was used to create the grid maps with a spacing of 0.375 Å. All molecular interactions were visualized by the Discovery Studio module of Biovia software [97].

#### 3.4.4. ADMET Properties

For the purpose of identifying significant compounds that exhibit drug-likeness for current in silico studies, excellent and better experimental ADMET (absorption, distribution, metabolism, excretion, and toxicity) features are required. The physicochemical properties of the compounds under study, including MW, nHBA, nHBD, and Log P were estimated. Moreover, the properties in accordance with absorption and distribution included volume of distribution, HIA, Caco2 permeability, and BBB. Metabolism-associated properties were estimated using CYP2C19 inhibitor. Excretion included clearance. Toxicity was evaluated based on AMES toxicity. All the parameters were computed using the online server ADMET lab 2.0 [98].

### 3.5. DFT Studies

To thoroughly investigate the molecular structures of the molecules under study, density functional theory (DFT) calculations were executed at the B3LYP/6-31G level using Gaussian09 software [99]. The geometries of the studied compounds were first optimized and further submitted to frequency computations to ensure if the obtained structures were true minima or not. The distributions and energies of the highest occupied molecular orbital (HOMO) and the lowest unoccupied molecular orbital (LUMO) were unveiled by means of the Frontier molecular orbitals (FMO). To well-characterize the features of the studied compounds, a diversity of global reactivity descriptors, including global hardness (*ƞ*), global softness (*σ*), polarizability (*α*), and dipole moment (*μ*), was assessed.

## 4. Conclusions

A series of 2-methyl-5-(2-phenylquinolin-4-yl)-1,3,4-oxadiazole were synthesized with various substituents and analyzed against monoamine oxidase (MAO-A and MAO-B) and acetylcholinesterase (AChE) enzymes. All molecules showed promising inhibition in the lower μM range against targeted enzymes. Compounds **5a** and **5f** were the most promising MAO-A and MAO-B inhibitors (IC_50_ = 0.91 ± 0.15 and 0.84 ± 0.06 nM, respectively). While compound **5c** exhibited the most efficient acetylcholinesterase inhibition (IC_50_ = 1.02 ± 0.65 μM). Furthermore, **5a**–**f** and **6a**–**d** were in silico investigated towards MAO-A, MAO-B, and AChE as anti-Alzheimer treatments using the AutoDock4.2.6 software. According to docking scores results, **5a**, **5f**, and **5c** demonstrated the promising docking scores against the investigated enzymes. Moreover, the crucial function played by the investigated enzymes in Alzheimer’s remediation proposes that promising molecules may act as potent novel chemical entities in identifying multi-target-directed inhibitors.

## Figures and Tables

**Table 1:** IC_50_ values of synthesized compounds towards MAO-A, MAO-B, and AChE enzymes.

No	Compound	IC_50_ (μM)		
		MAO-A	MAO-B	AChE
1	**5a**	0.91 ± 0.15	1.59 ± 1.66	1.40 ± 0.45
2	**5b**	1.81 ± 0.38	2.61 ± 2.48	2.55 ± 0.96
3	**5c**	3.31 ± 0.80	3.39 ± 0.42	1.02 ± 0.65
4	**5d**	3.18 ± 1.23	3.76 ± 1.04	2.38 ± 0.92
5	**5e**	4.14 ± 0.35	3.84 ± 0.91	1.29 ± 0.75
6	**5f**	4.88 ± 1.75	0.84 ± 0.06	3.32 ± 0.45
7	**6a**	1.51 ± 0.52	3.71 ± 2.88	3.23 ± 0.95
8	**6b**	1.02 ± 0.92	2.71 ± 0.88	3.54 ± 1.05
9	**6c**	6.81 ± 2.65	5.59 ± 3.22	4.38 ± 1.45
10	**6d**	4.16 ± 1.71	2.90 ± 1.85	4.98 ± 1.85
11	Clorgyline ^b^	0.0045 ± 0.0003	61.35 ± 1.13	
12	Deprenyl ^b^	67.25 ± 1.02	0.0196 ± 0.001	
13	Donepezil ^b^			0.032 ± 0.003 ^a^

^a^ All data are expressed as the average ± SEM of determinations made in triplicate. ^b^ Standard inhibitors.

**Table 2:** Drug-like and ADMET characteristics of potent synthesized molecules and controls ^a^.

Compound	Physicochemical Properties					ADMET Properties					
						Absorption and Distribution			Metab-Olism (CYP2C19 Inhibitor)	Excretion (Clearance)	Toxicity (AMES)
	MW	nHBA	nHBD	Log P	Lipinski Rule	HIA	CaCo-2	BBB			
**5a**	464.1	7	0	5.2	Accepted	0.007	−4.55	0.058	0.889	3.10	0.043
**5c**	492.1	7	0	5.4	Accepted	0.006	−4.534	0.078	0.862	3.22	0.012
**5f**	534.2	7	0	6.7	Accepted	0.006	−4.582	0.050	0.819	3.46	0.011
Clorgyline	271.1	2	0	3.7	Accepted	−0.001	−4.251	0.992	0.679	11.24	0.017
Deprenyl	187.1	1	0	2.7	Accepted	−0.005	−4.915	0.996	0.133	10.46	0.035
Donepezil	379.2	4	0	4.2	Accepted	0.003	−4.793	0.975	0.413	10.63	0.026

^a^ MW: Molecular weight; nHBA: number of hydrogen bond acceptors; nHBD: number of hydrogen bond donors; Log P: log of the octanol-water partition coefficient; HIA: Human Intestinal Absorption; CaCo-2: CaCo-2 permeability; BBB: Blood Brain Barrier.

**Table 3:** Energies of the optimized compounds (*E*_opt_, in au), the highest occupied molecular orbital (*E*_HOMO_, in eV), and the lowest unoccupied molecular orbital (*E*_LUMO_, in eV), along with energy gap (*E*_gap_, in eV). Computed values of global hardness (*ƞ*), global softness (*σ*), polarizability (*α*), and dipole moment (*μ*) of the studied compounds.

Compound	*E*_opt_ (au)	*E*_HOMO_ (eV)	*E*_LUMO_ (eV)	∆*E*_gap_ (eV)	*η* (eV)	*σ* (eV^−1^)	*μ* (Debye)	*α*
**5a**	−1842.831	−0.221	−0.112	0.108	0.054	9.206	8.358	347.284
**5b**	−1882.144	−0.221	−0.109	0.112	0.056	8.917	7.599	351.377
**5c**	−1921.445	−0.226	−0.100	0.125	0.063	7.955	6.751	363.499
**5d**	−1960.751	−0.226	−0.098	0.127	0.064	7.846	7.320	370.377
**5e**	−2000.055	−0.225	−0.096	0.129	0.065	7.721	6.769	385.049
**5f**	−2039.360	−0.225	−0.095	0.130	0.065	7.651	7.268	393.625
**6a**	−1899.057	−0.227	−0.089	0.137	0.069	7.266	1.194	331.243
**6b**	−1899.057	−0.227	−0.088	0.138	0.069	7.237	2.973	329.504
**6c**	−1899.059	−0.226	−0.088	0.138	0.069	7.245	2.446	323.636
**6d**	−1562.098	−0.223	−0.084	0.138	0.069	7.204	3.093	318.962

## Data Availability

The data presented in this study are available in the Supplementary Material.

## Supplementary Materials

The following supporting information can be downloaded at: https://www.mdpi.com/article/10.3390/ph16010011/s1, Figure S1: ^1^H-NMR, ^13^C-NMR, and FT-IR Spectra of compounds **5a**–**f** and **6a**–**d**.
