# Supplementary material for: Synthesis and Evaluation of Novel S-alkyl Phthalimide- and S-benzyl-oxadiazole-quinoline Hybrids as Inhibitors of Monoamine Oxidase and Acetylcholinesterase
Source: Pharmaceuticals (Basel). 2022 Dec 22;16(1):11. doi: 10.3390/ph16010011 (PMC9865589; doi:10.3390/ph16010011)

Supplementary Material

^{1}H-NMR, ^{13}C-NMR and FT-IR Spectra of ccompounds5a-f and 6a-d

^{1}H-NMR Spectra of 5a

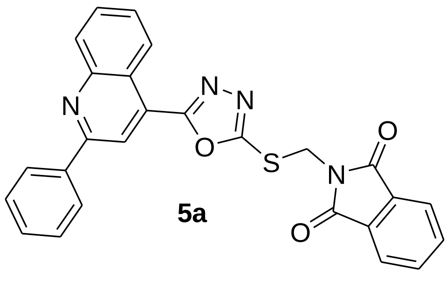

5a

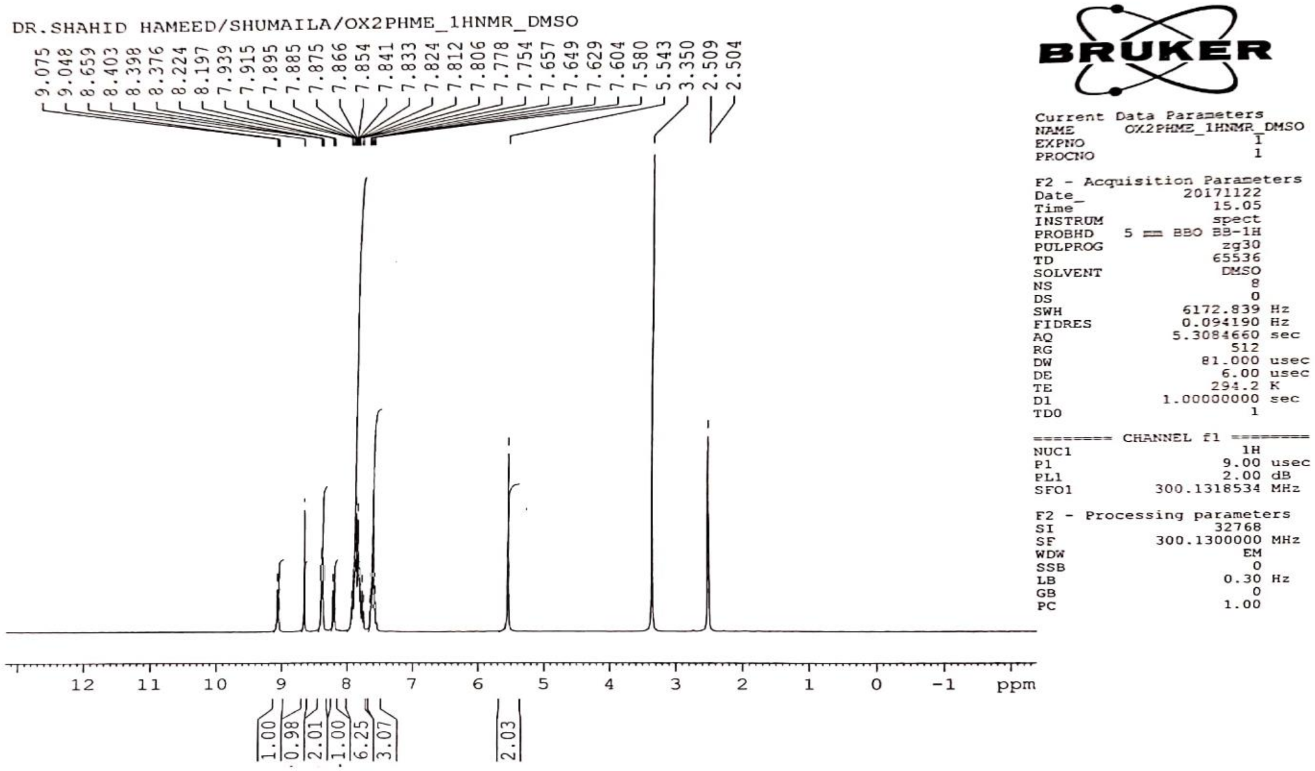

13C-NMR Spectra of 5a

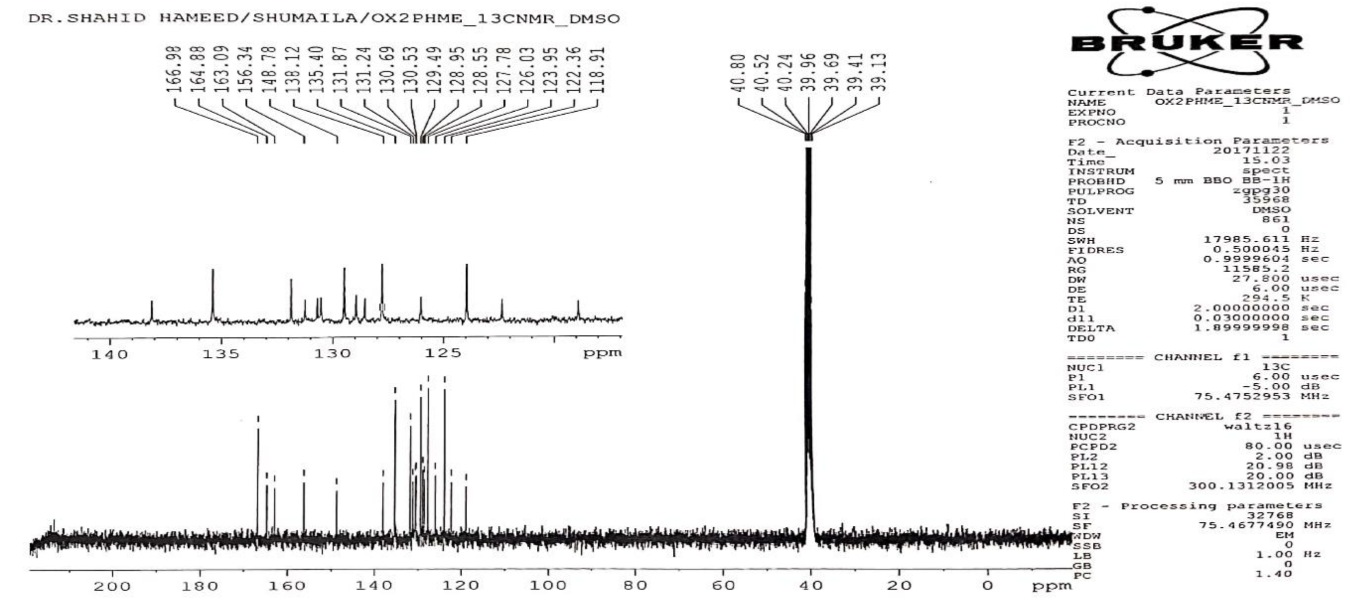

FT-IR Spectra of 5a

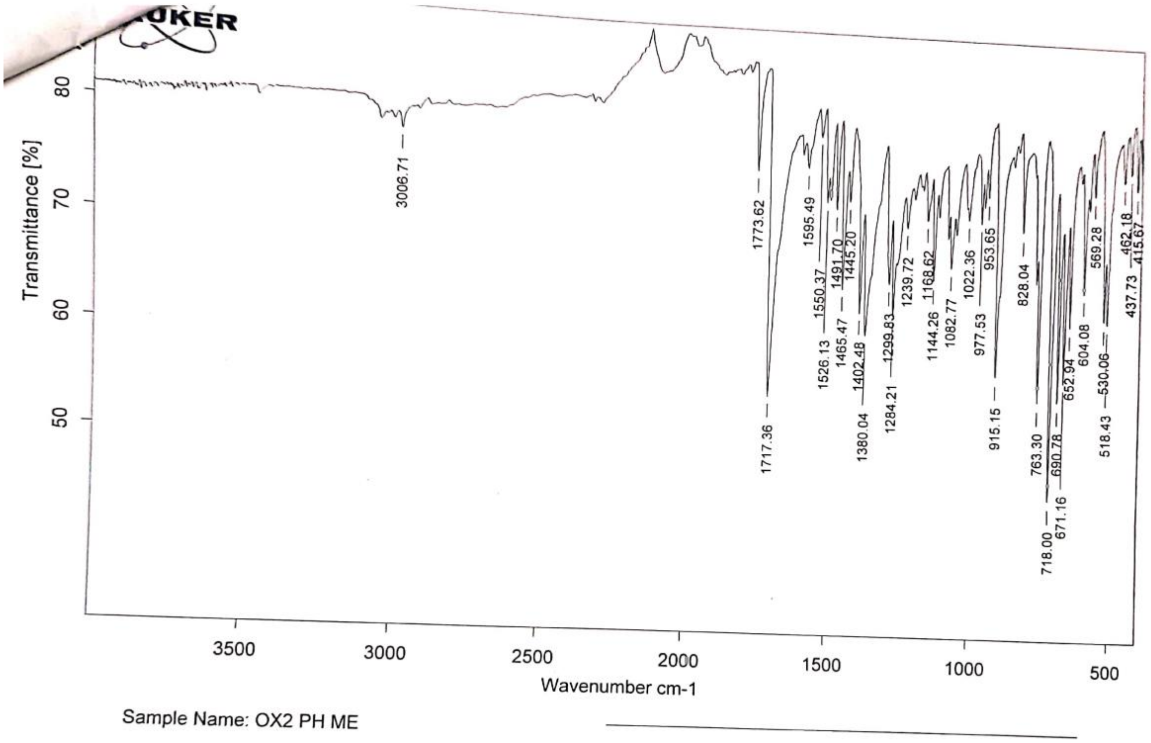

Scanned with CamScanner

^{1}H-NMR Spectra of 5b

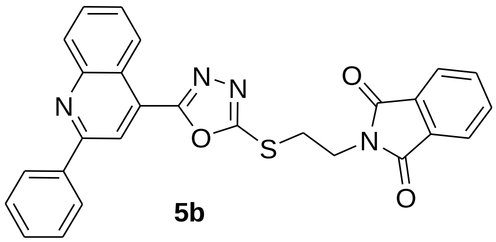

5b

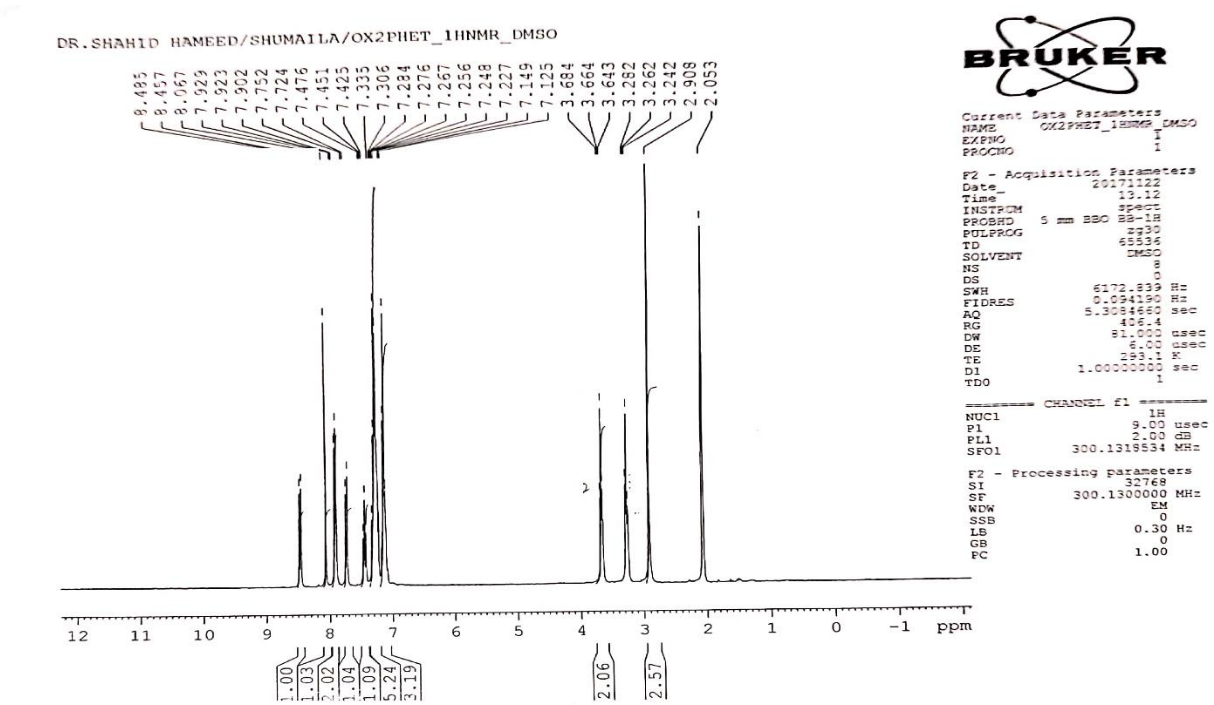

^{13}C-NMR Spectra of 5b

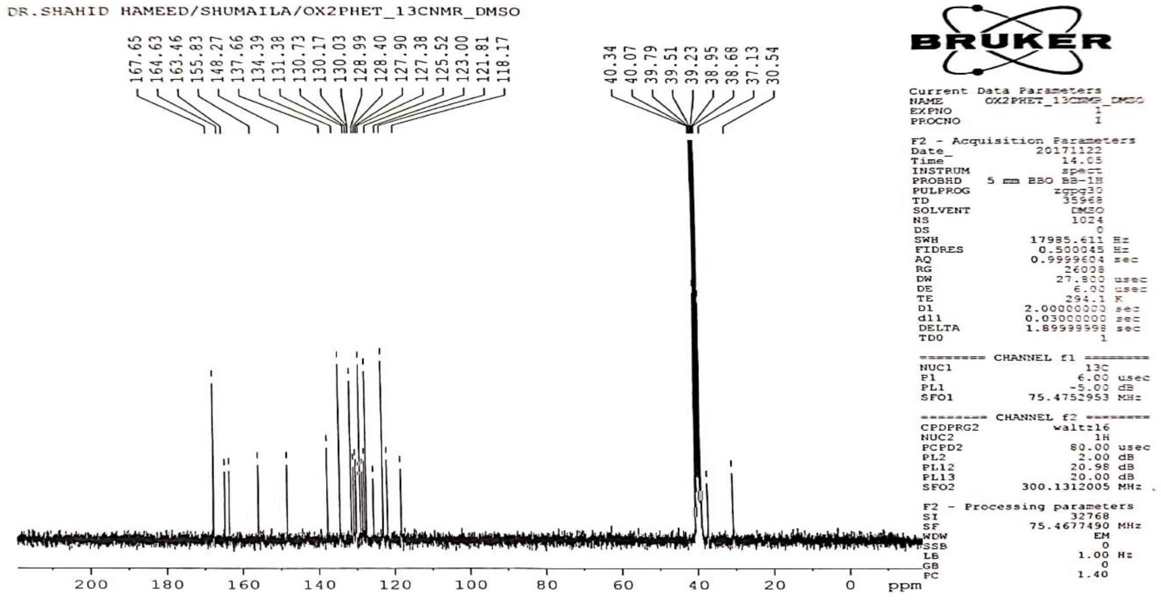

FT-IR Spectra of 5b

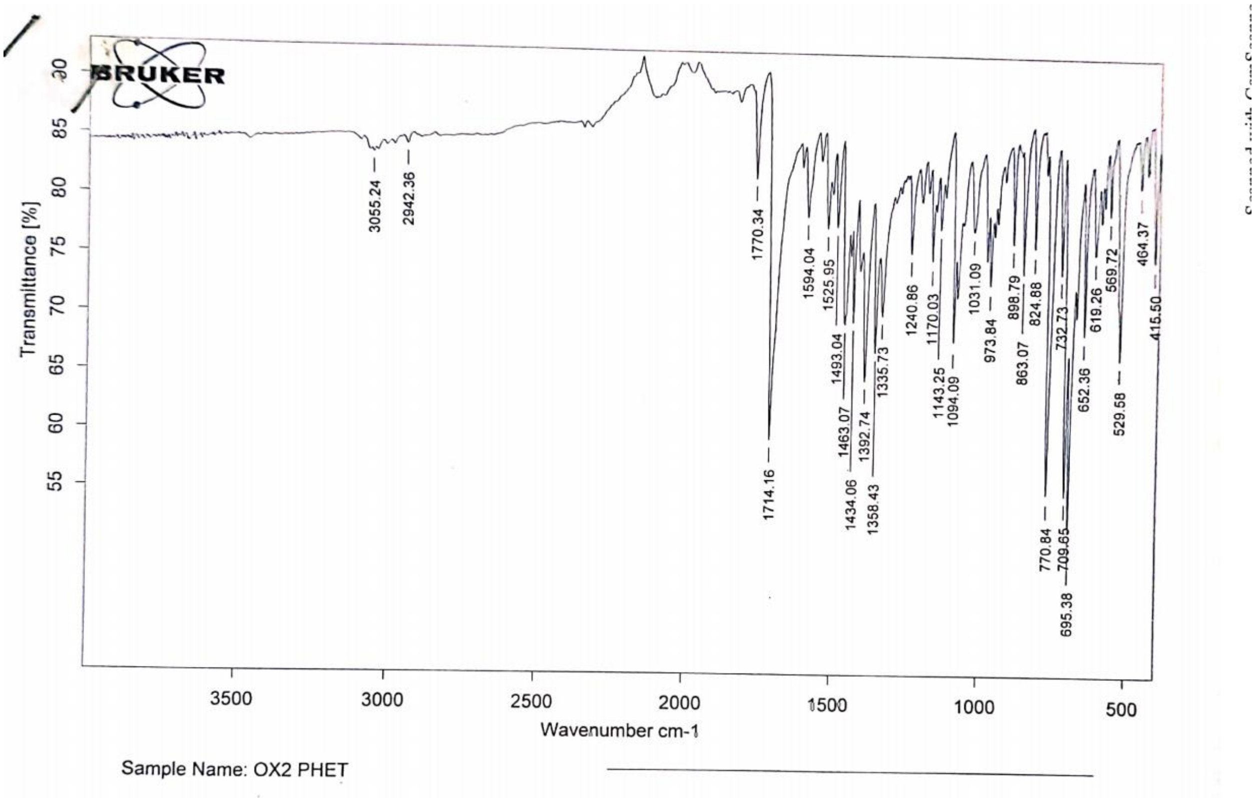

^{1}H-NMR Spectra of 5c

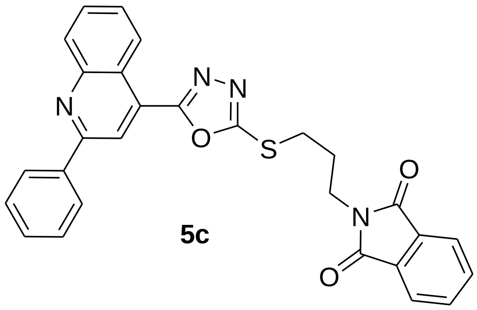

5c

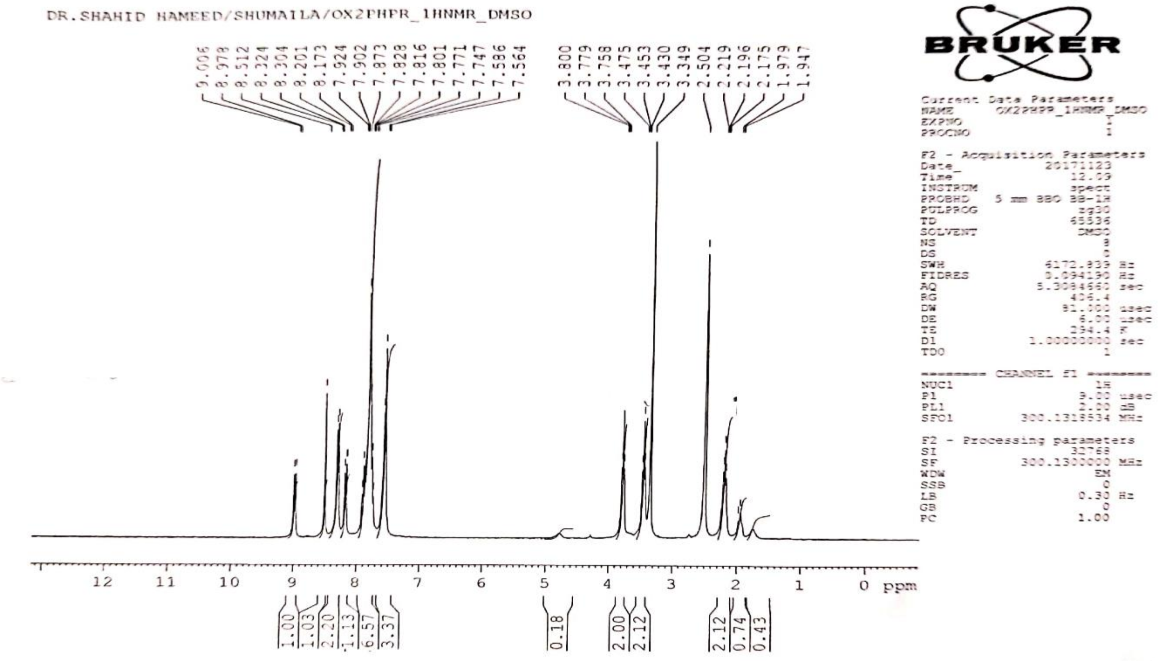

13C-NMR Spectra of 5c

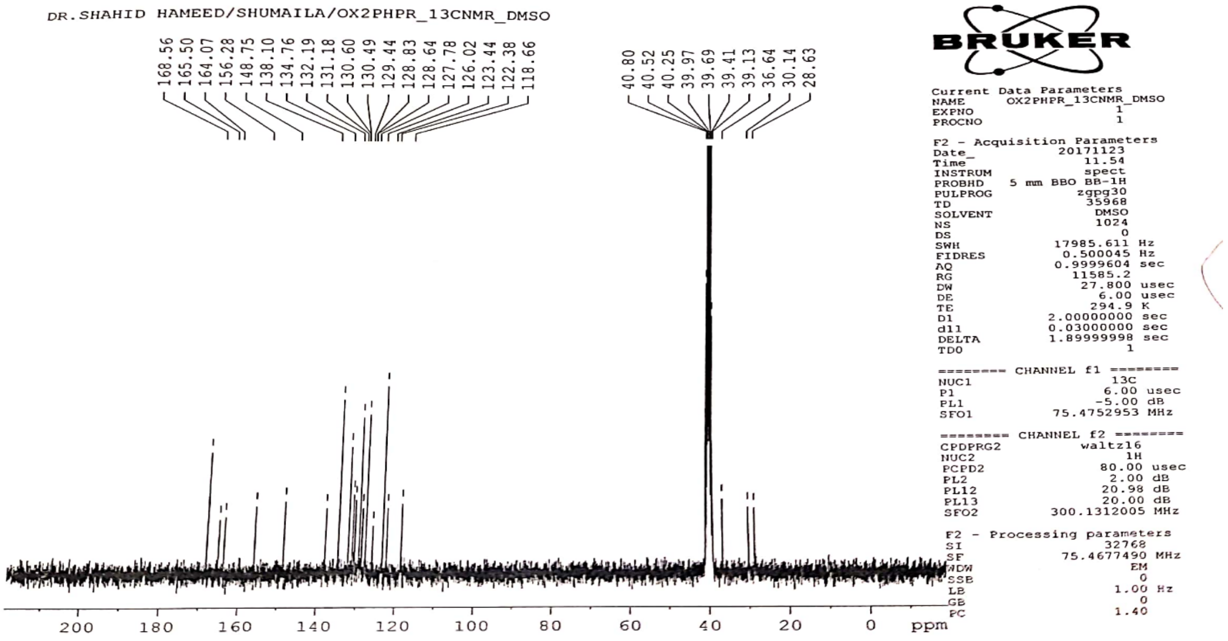

FT-IR Spectra of 5c

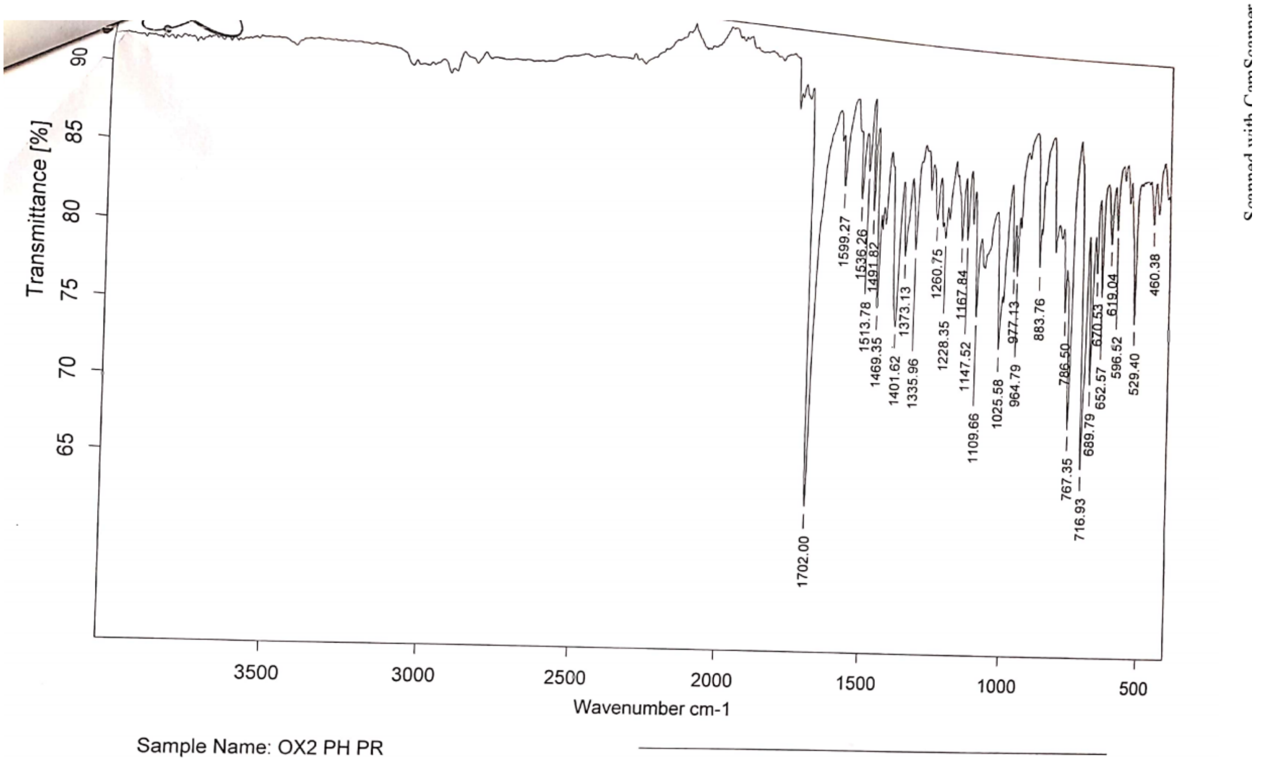

^{1}H-NMR Spectra of 5d

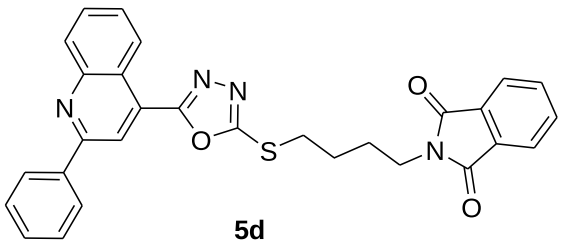

5d

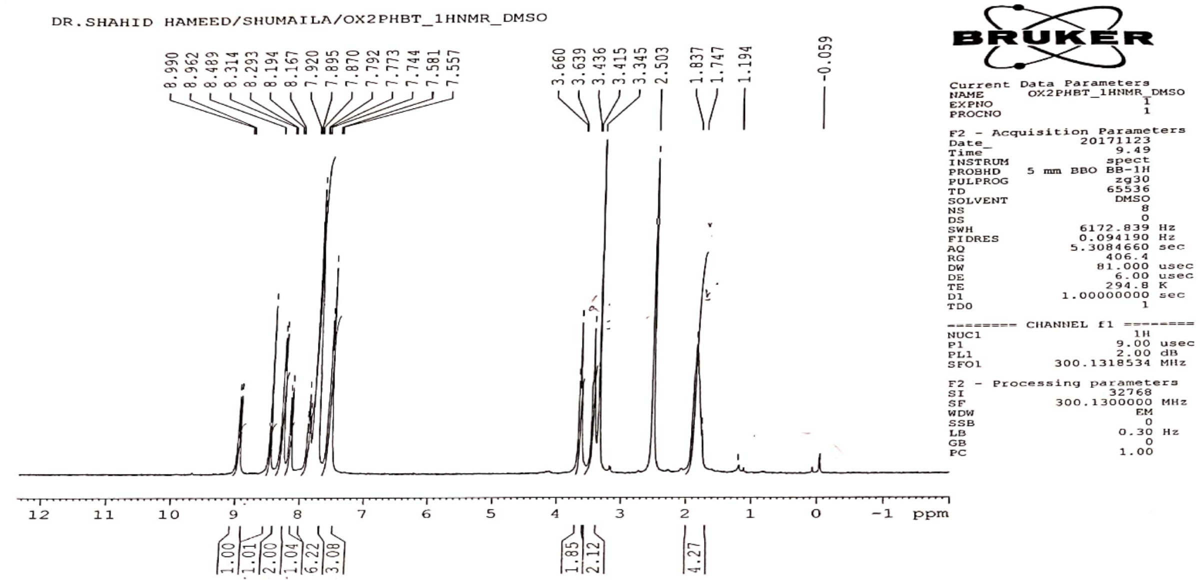

^{13}C-NMR Spectra of 5d

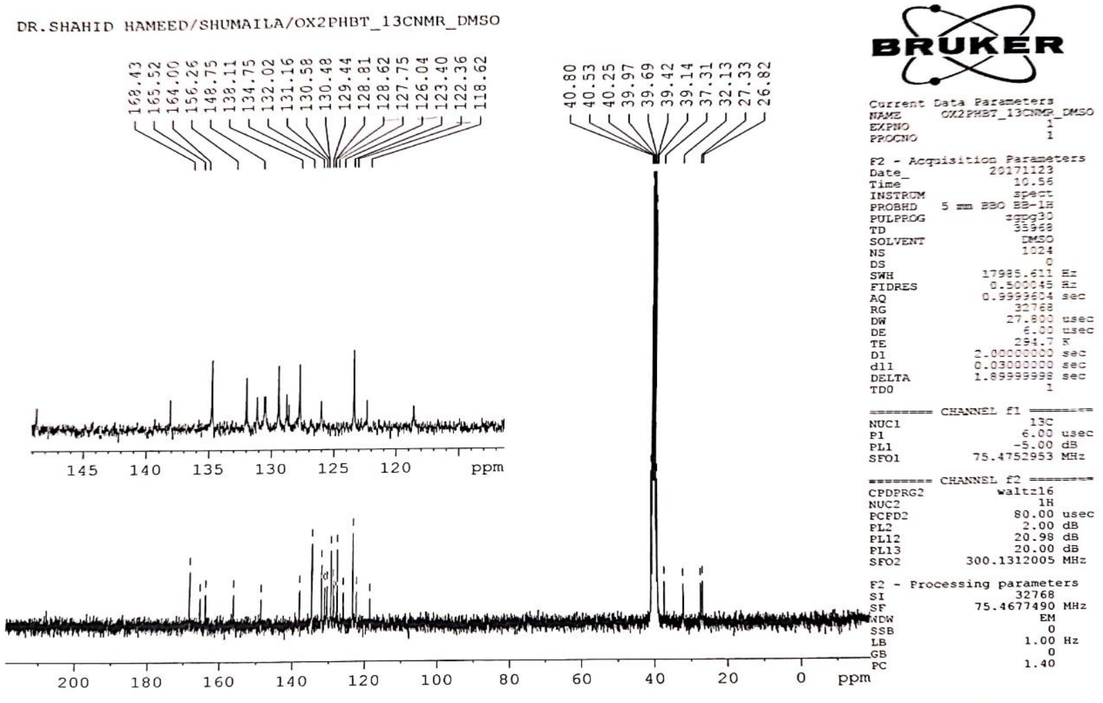

FT-IR Spectra of 5d

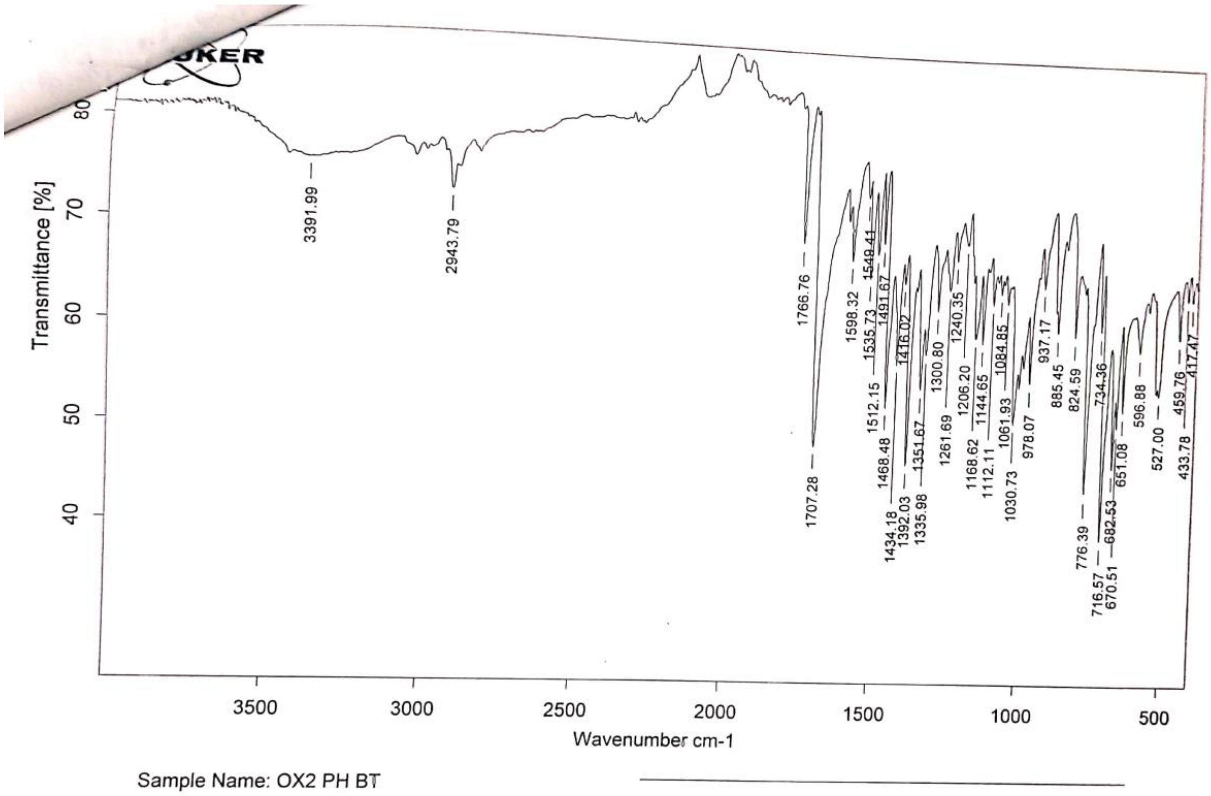

^{1}H-NMR Spectra of 5e

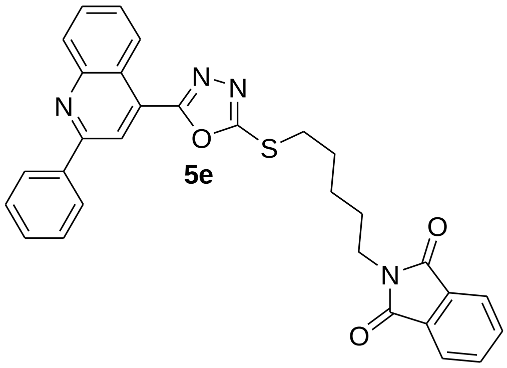

5e

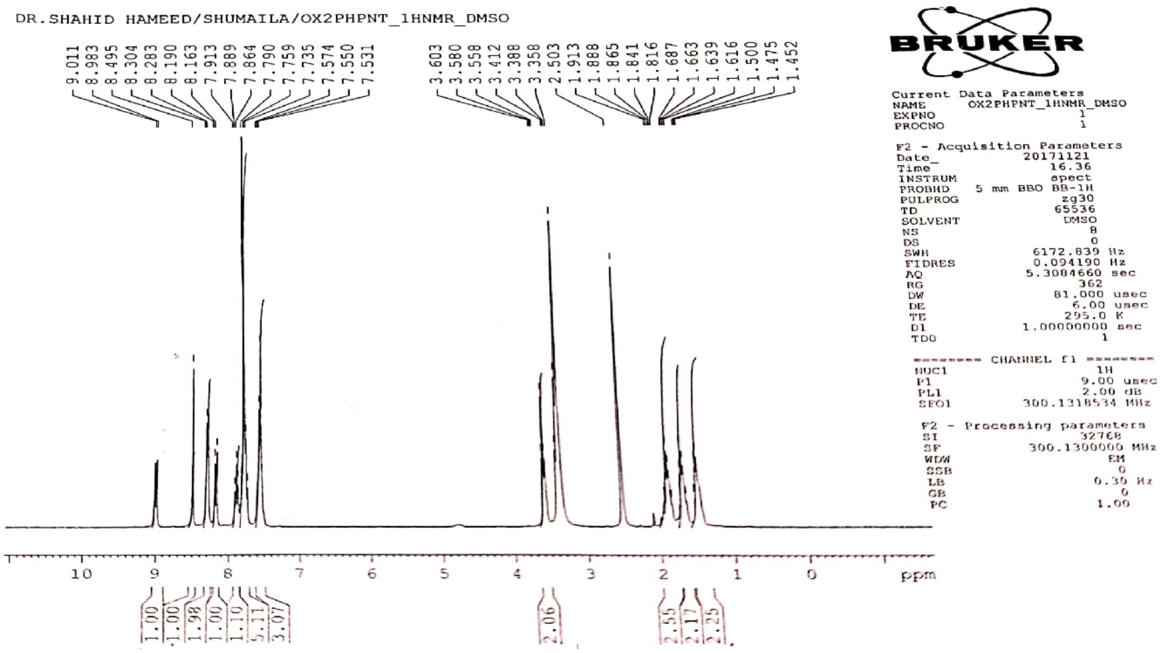

^{13}C-NMR Spectra of 5e

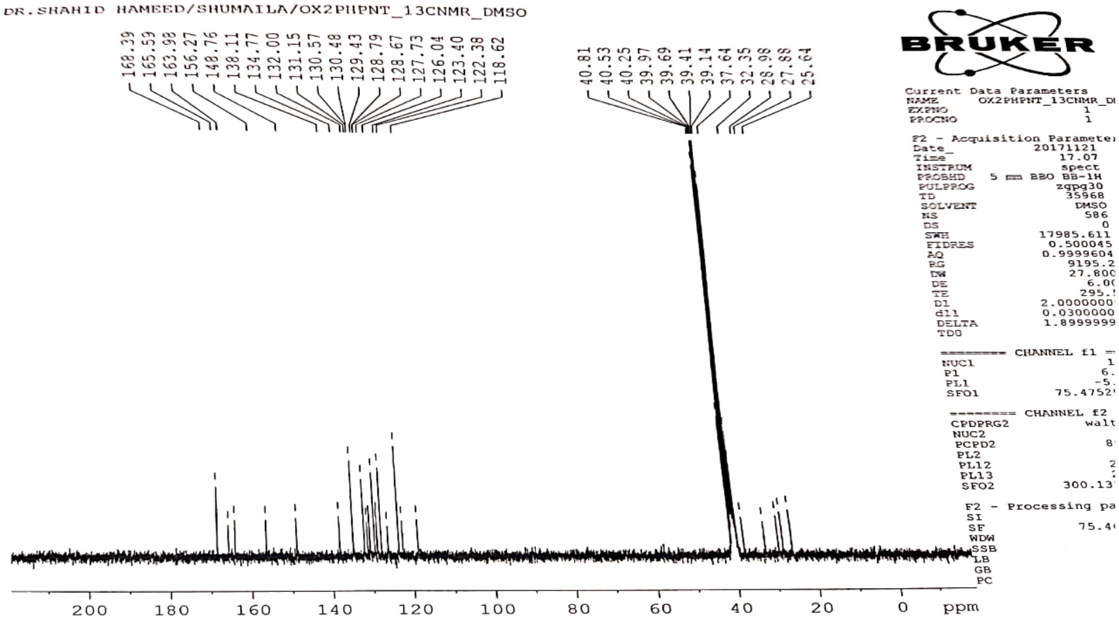

FT-IR Spectra of 5e

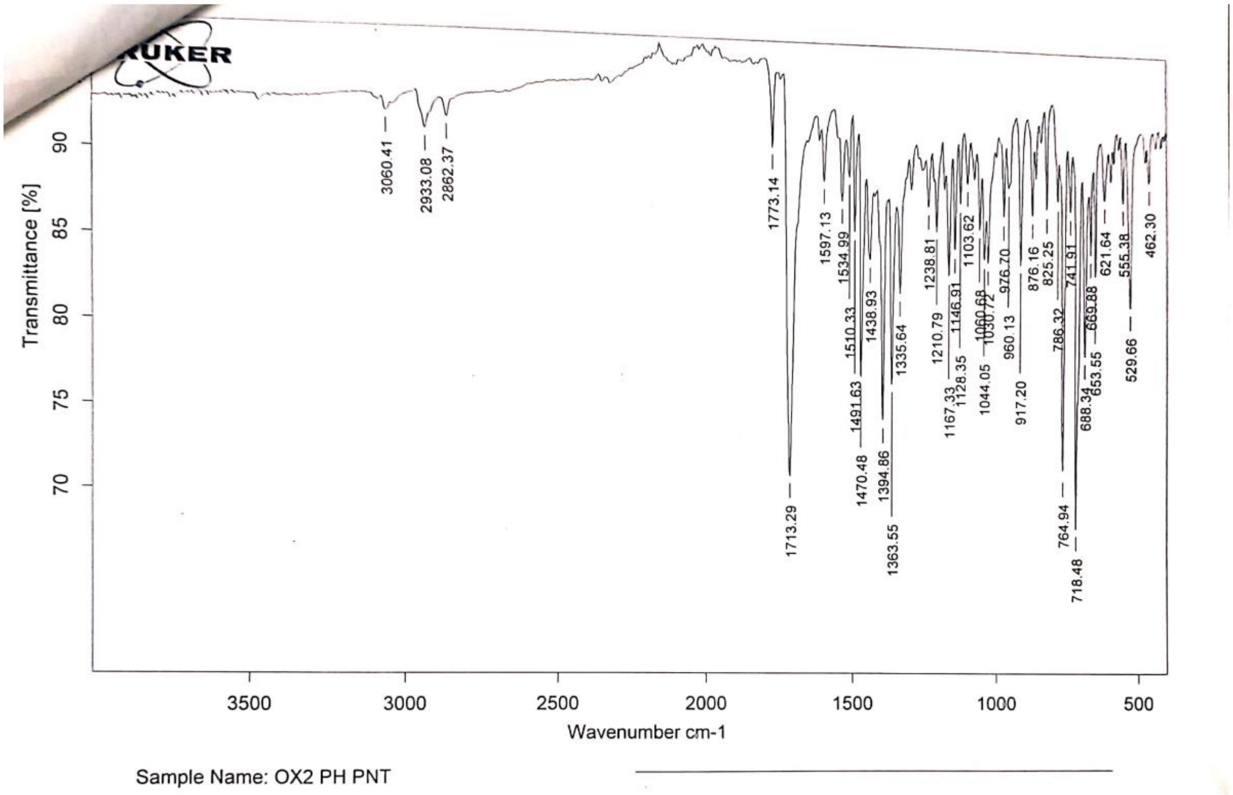

^{1}H-NMR Spectra of 5f

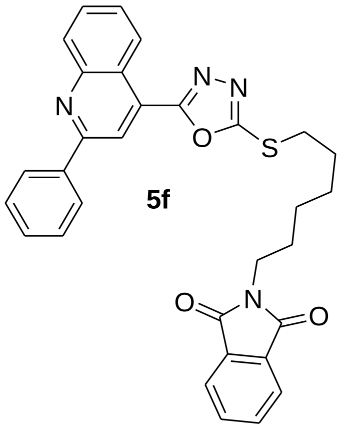

5f

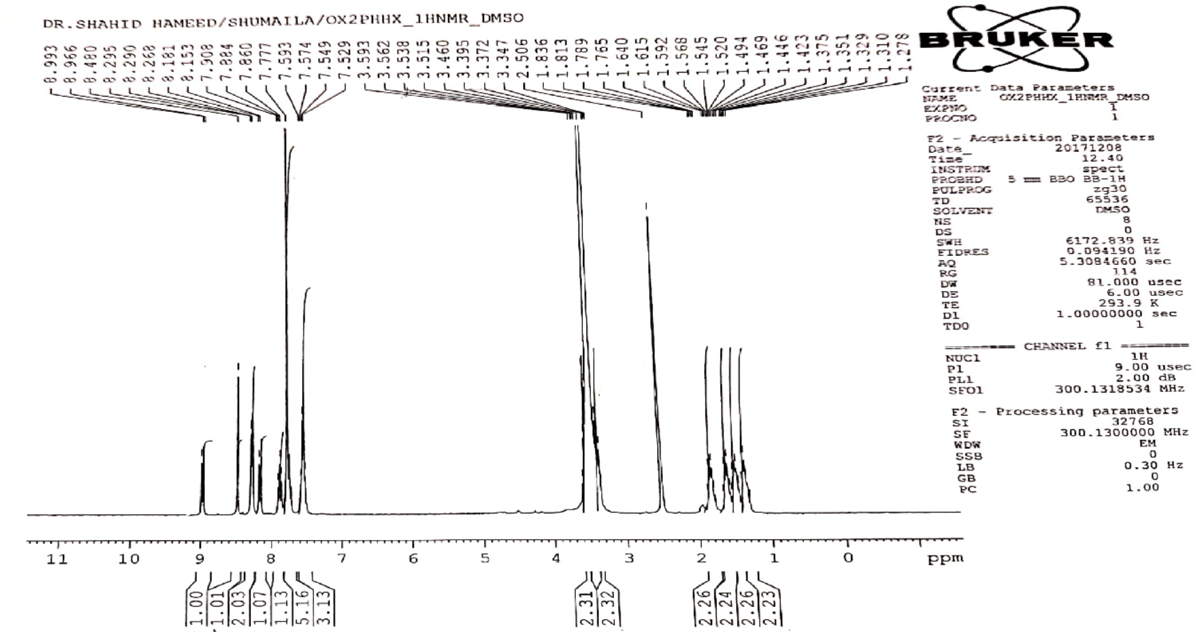

^{13}C-NMR Spectra of 5f

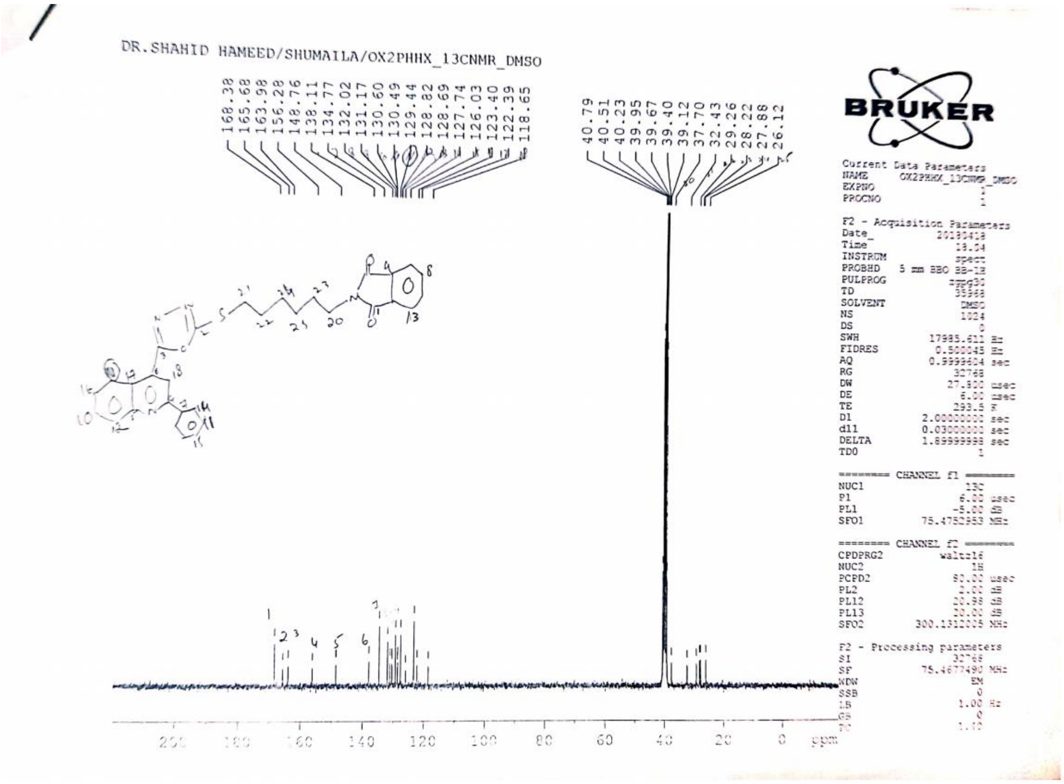

FT-IR Spectra of 5f

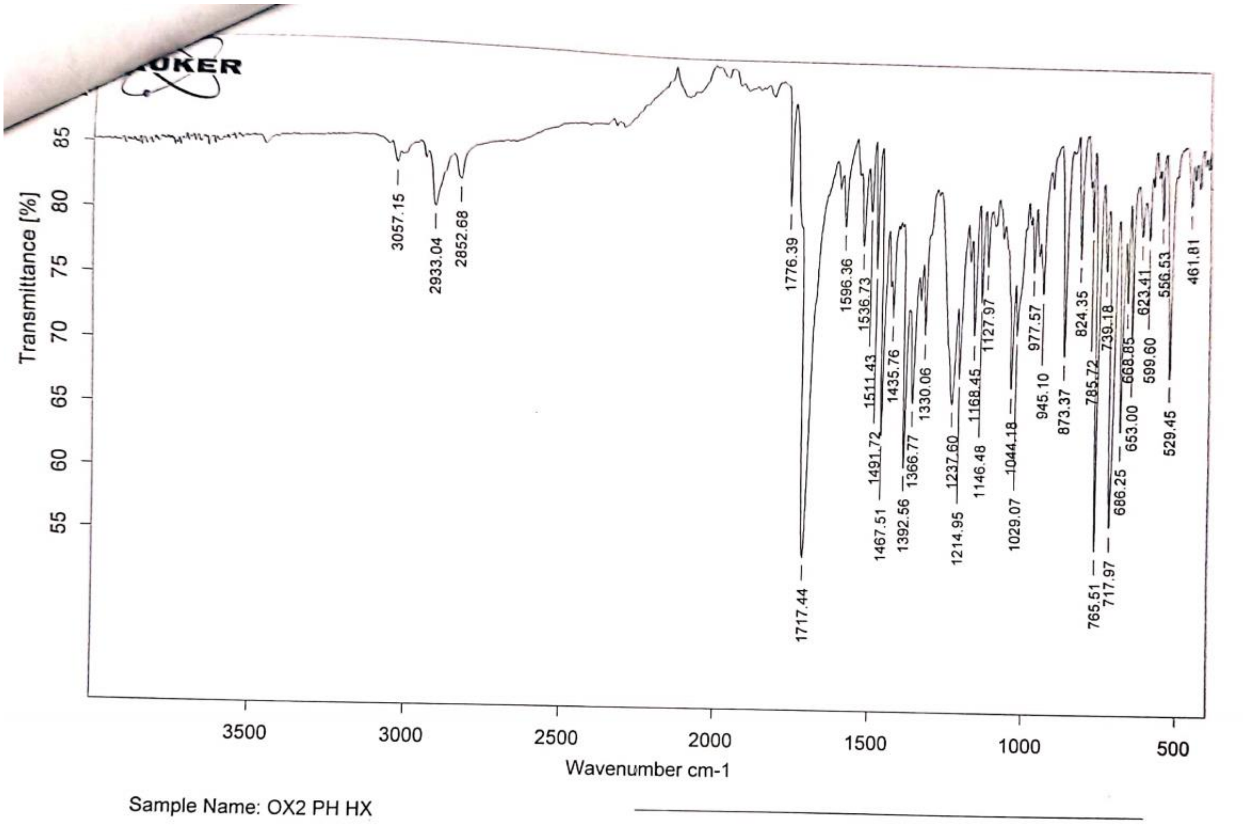

1H-NMR Spectra of 6a

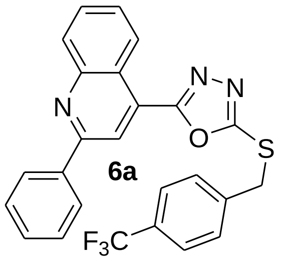

6a

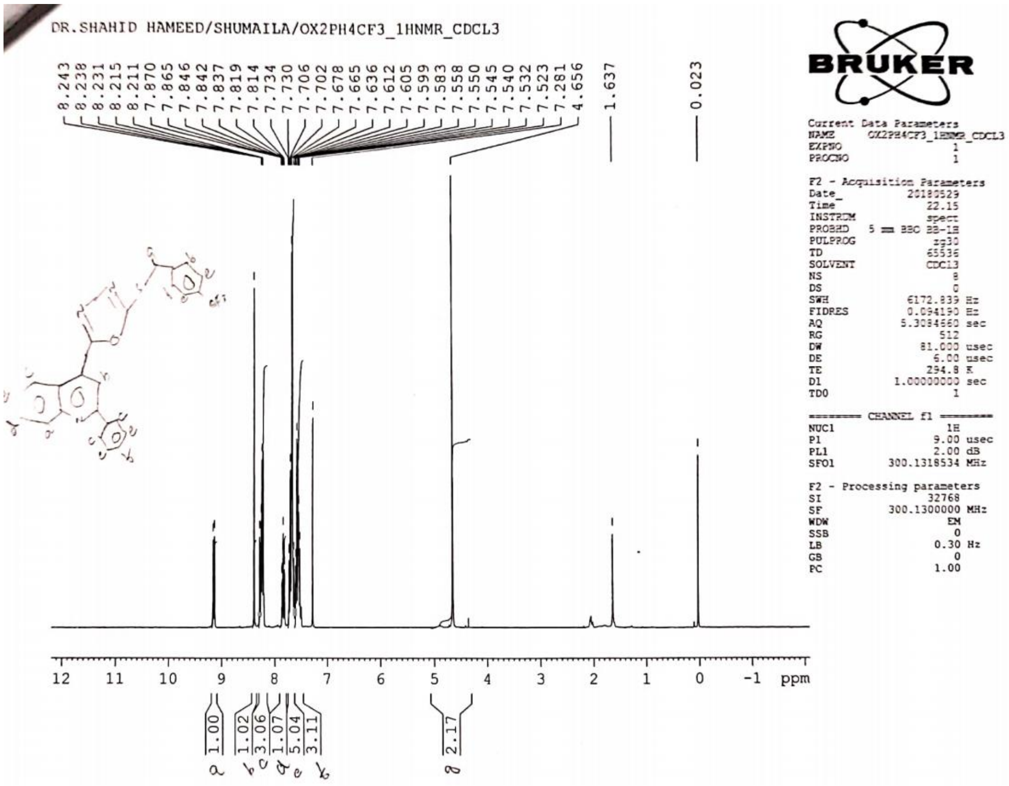

13C-NMR Spectra of 6a

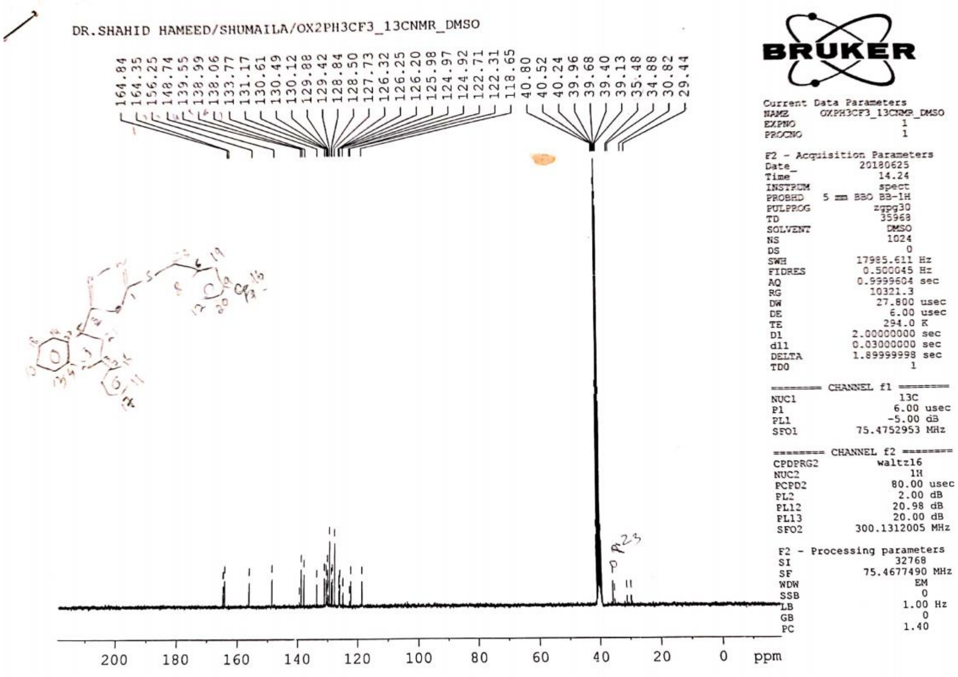

FT-IR Spectrum of 6a

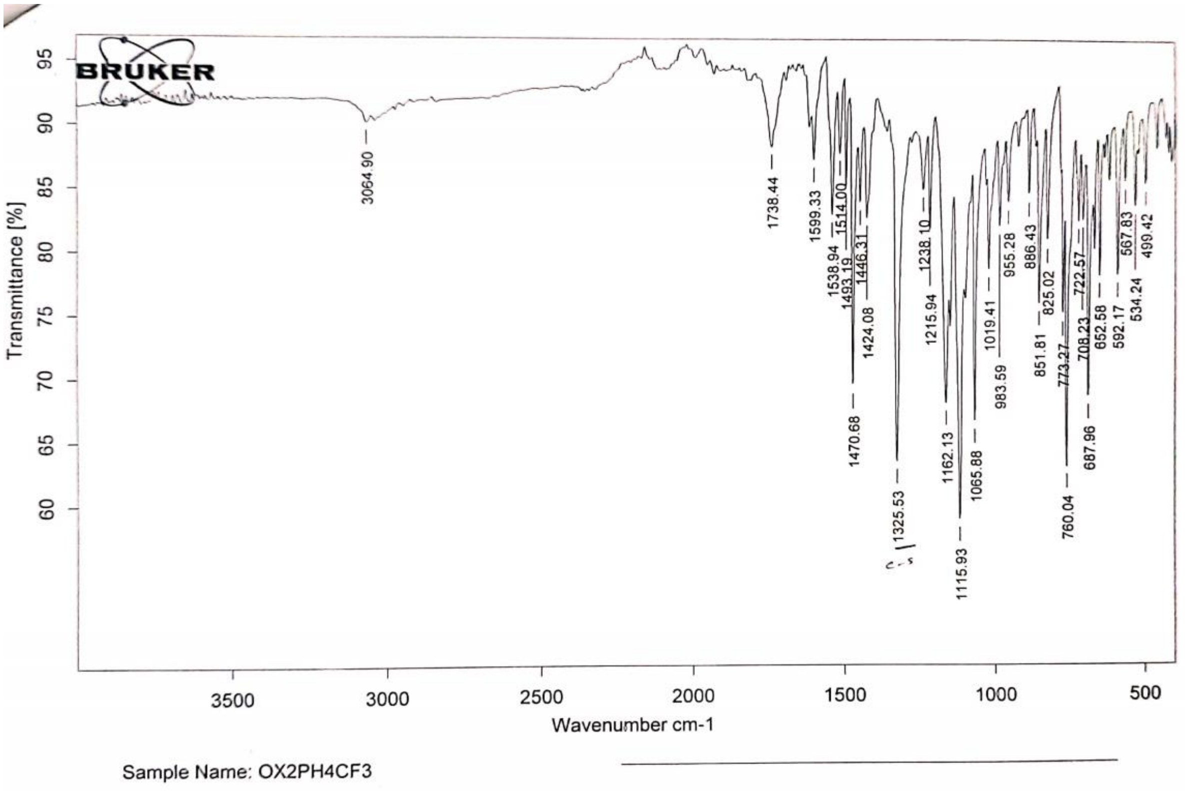

1H-NMR Spectra of 6b

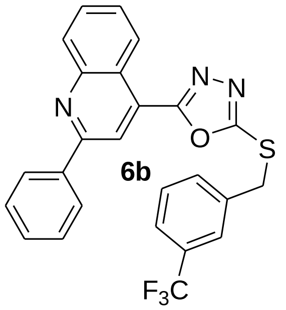

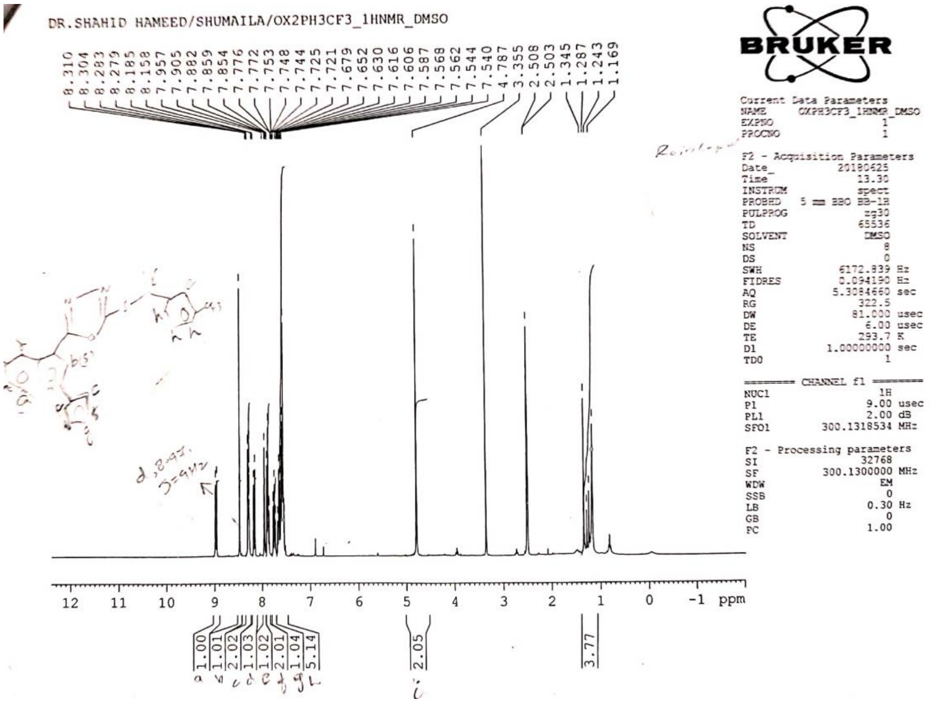

13C-NMR Spectra of 6b

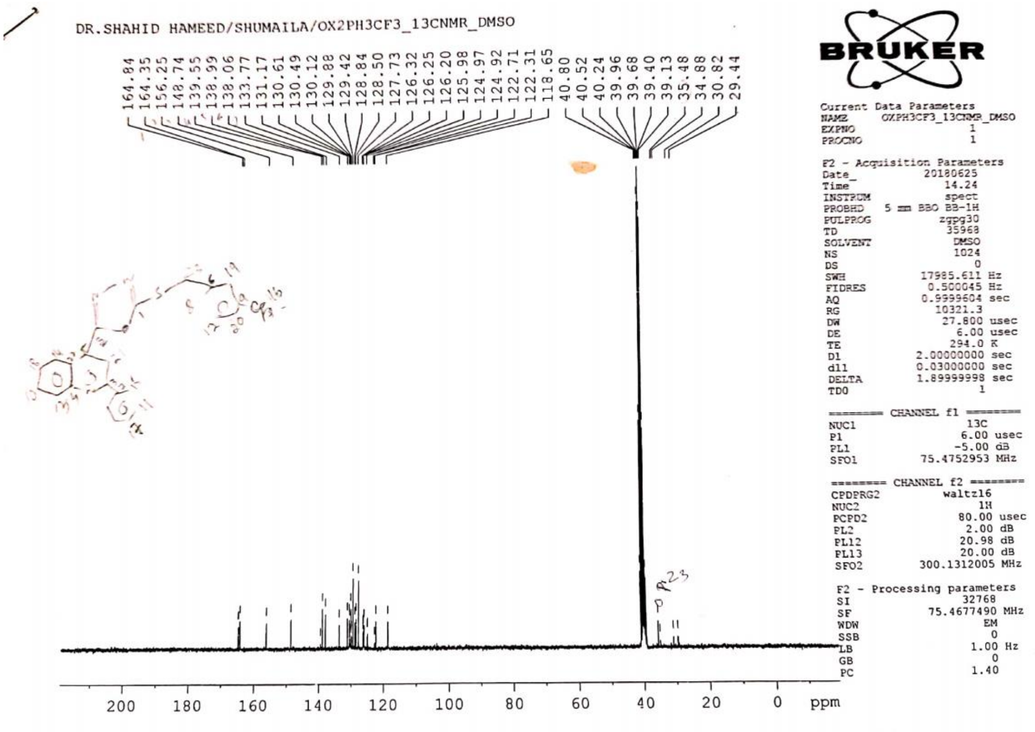

FT-IR Spectra of 6b

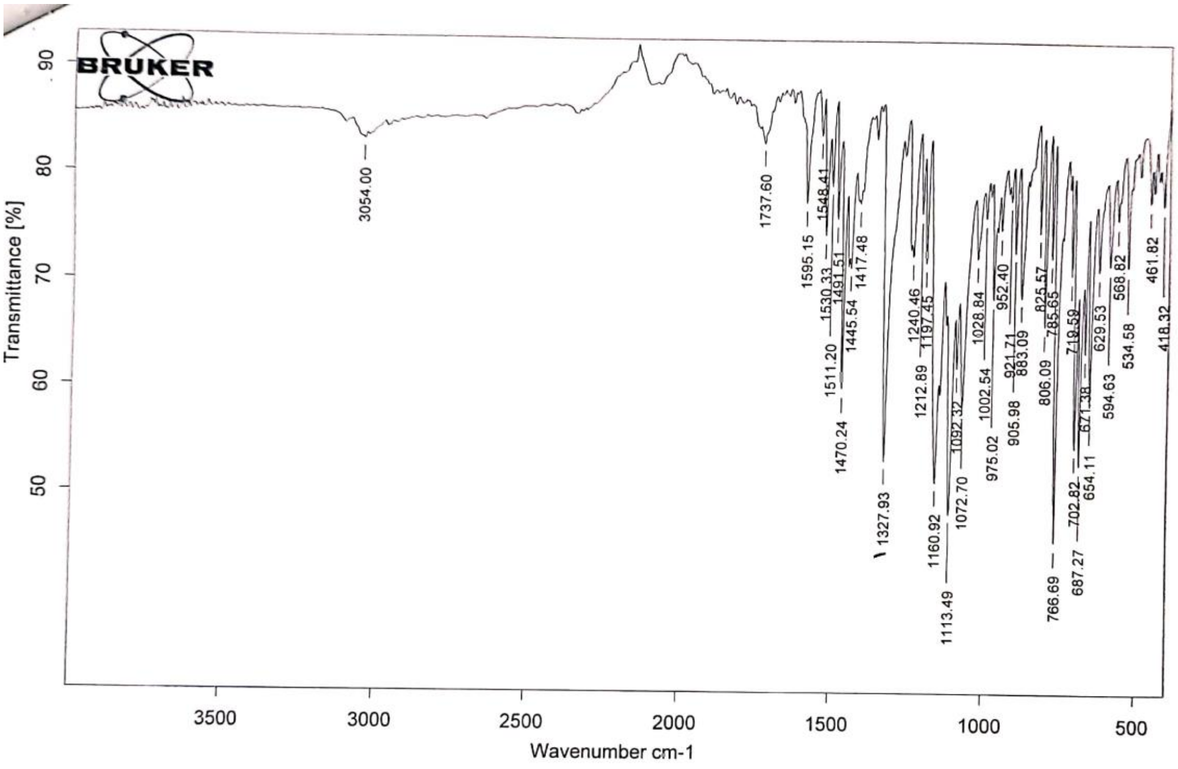

Sample Name: OX2PH3CF3

1H-NMR Spectra of 6c

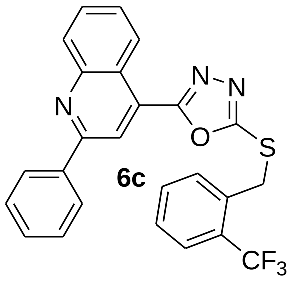

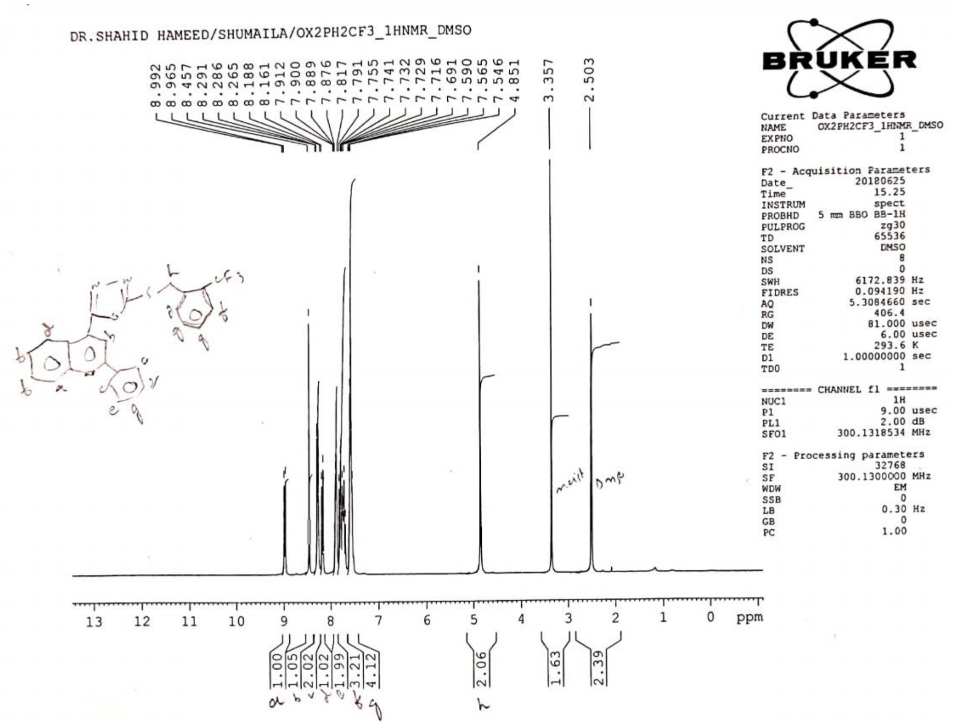

13C-NMR Spectra of 6c

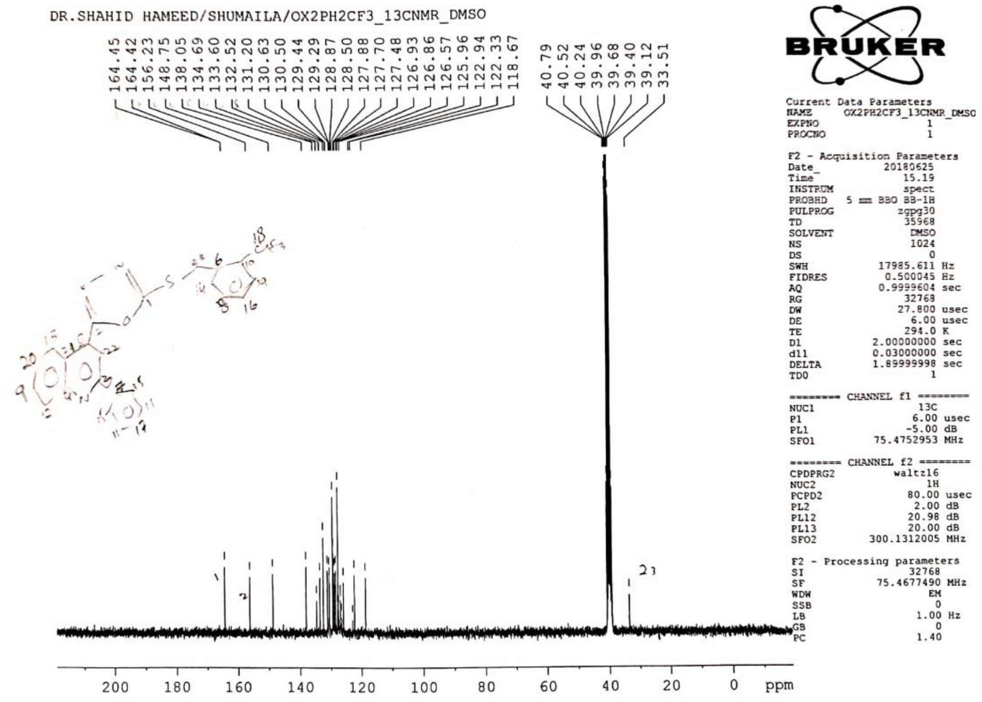

FT-IR Spectra of 6c

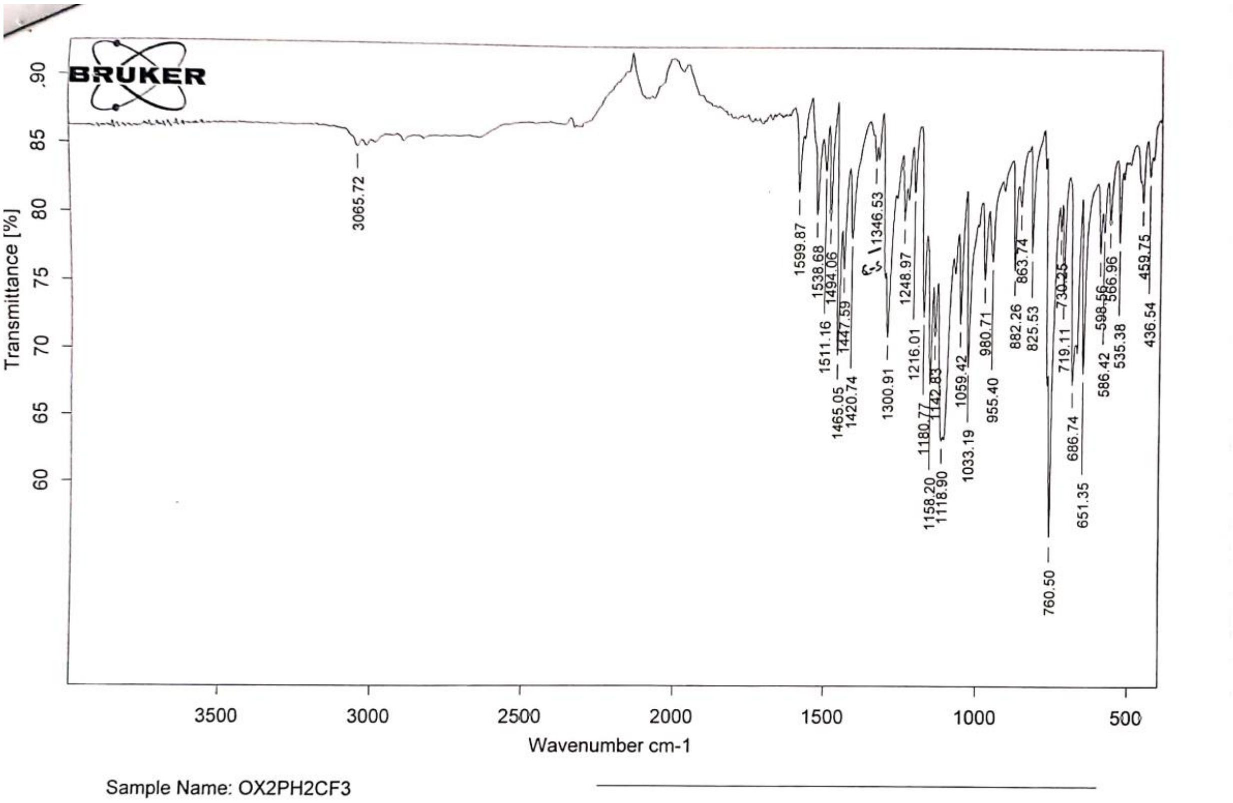

1H-NMR Spectra of 6d

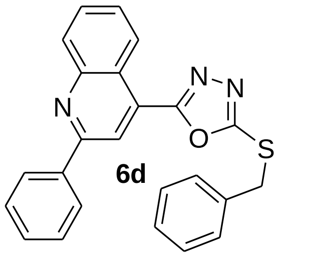

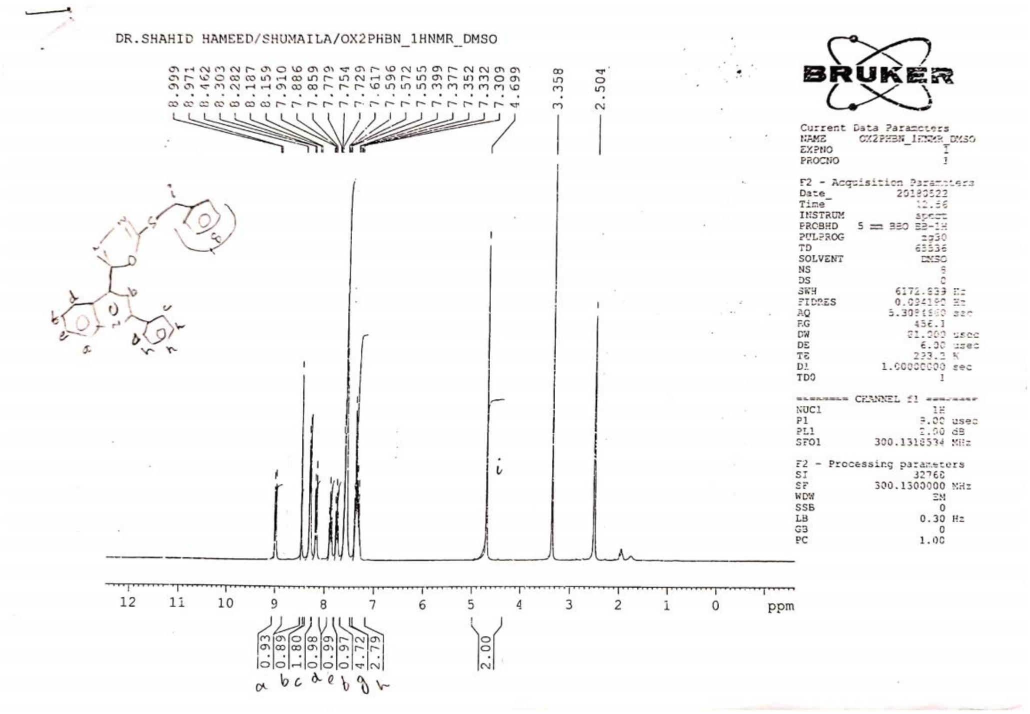

13C-NMR Spectra of 6d

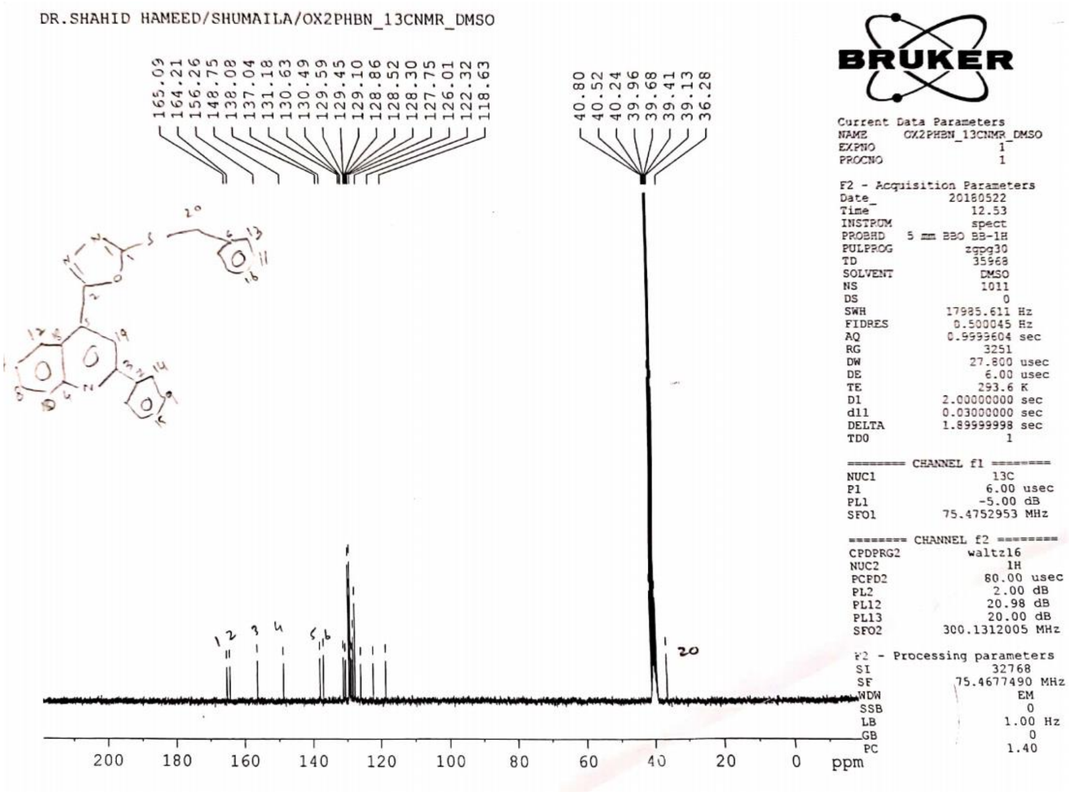

FT-IR Spectra of 6d

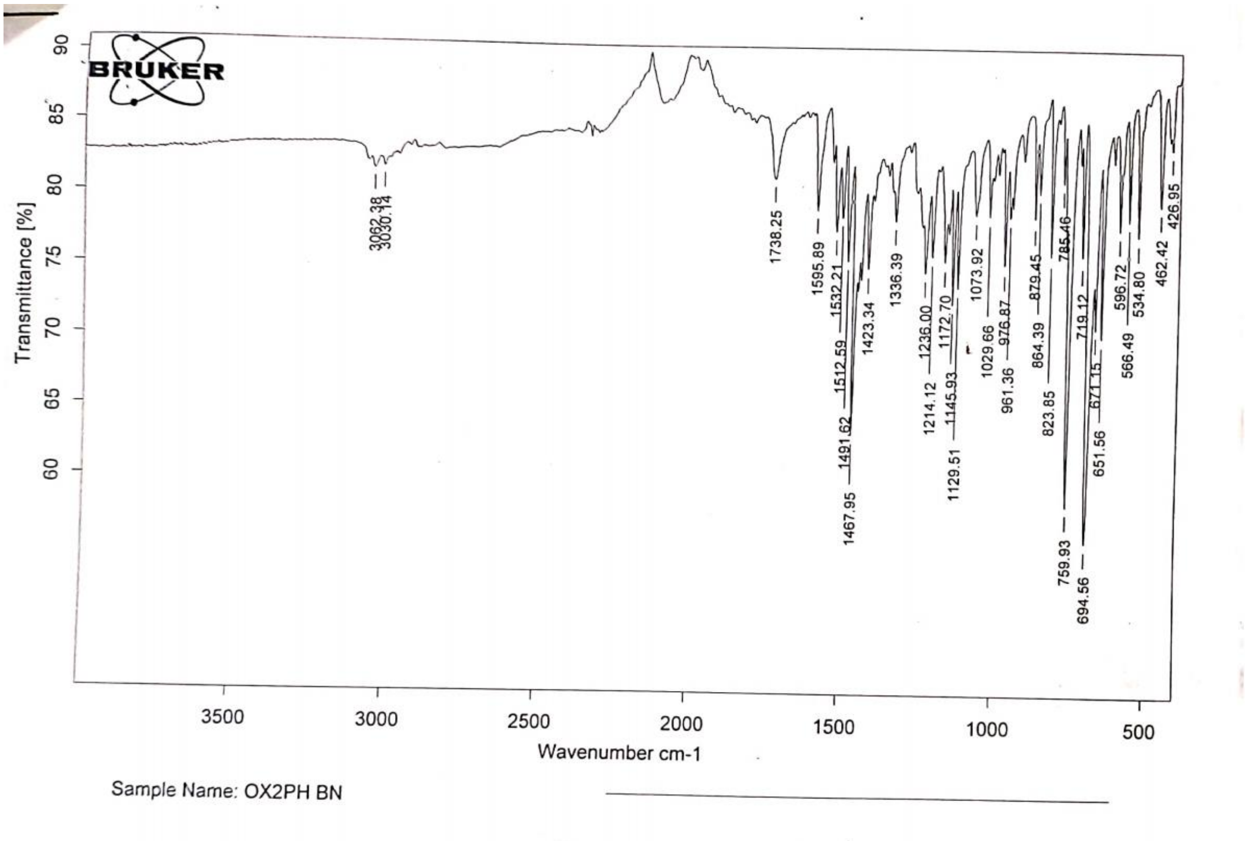

Supplement: Supplementary file 1 [file pharmaceuticals-16-00011-s001.zip › pharmaceuticals-2070849-supplementary.pdf]
